# Analysis of an event study using the Fama–French five-factor model: teaching approaches including spreadsheets and the R programming language

**DOI:** 10.1186/s40854-023-00477-3

**Published:** 2023-04-11

**Authors:** Monica Martinez-Blasco, Vanessa Serrano, Francesc Prior, Jordi Cuadros

**Affiliations:** 1 IQS School of Management-Universitat Ramon Llull, Via Augusta 390, 08017 Barcelona, Spain; 2 IQS School of Engineering-Universitat Ramon Llull, Via Augusta 390, 08017 Barcelona, Spain

**Keywords:** Event study, Fama–French five-factor model, Financial education, Teaching innovation, Spreadsheet, R programming language

## Abstract

The current financial education framework has an increasing need to introduce tools that facilitate the application of theoretical models to real-world data and contexts. However, only a limited number of free tools are available for this purpose. Given this lack of tools, the present study provides two approaches to facilitate the implementation of an event study. The first approach consists of a set of MS Excel files based on the Fama–French five-factor model, which allows the application of the event study methodology in a semi-automatic manner. The second approach is an open-source R-programmed tool through which results can be obtained in the context of an event study without the need for programming knowledge. This tool widens the calculus possibilities provided by the first approach and offers the option to apply not only the Fama–French five-factor model but also other models that are common in the financial literature. It is a user-friendly tool that enables reproducibility of the analysis and ensures that the calculations are free of manipulation errors. Both approaches are freely available and ready-to-use.

## Introduction

Financial education and massive data processing tools are two common requirements for a financial position (Yan 2017). Thus, finance students should be provided not only with the knowledge required to understand financial models but also with training in information gathering and analysis tools to apply financial models (Reese and Robins 2017). This study aims to demonstrate the limitations of information processing tools commonly used in finance classes, such as Microsoft Excel (MS Excel), by means of a real case study. We argue that more advanced data processing tools, such as R software, should be used in finance classes (Shi et al. 2017), as shown in the event study presented in this paper. Herrington et al. (2010) emphasized the importance of moving away from traditional classes and stressed on an approach to courses focusing on authentic tasks or projects to reflect how knowledge is applied in real life. Hence, real-life tasks may help promote and provide authentic learning (Herrington and Herrington 2006) in finance education. They help bridge the gap between theory and praxis, and include context-based knowledge. Learning seeks to deliver real-world experiences to students so that they can acquire the skills and competencies needed to succeed in today's workplace. We chose to illustrate how to move the event study methodology to a finance class using Excel- and R-based tools.

The event study methodology analyzes the market reaction to announcements of corporate events or news (Fehrs 1990). Recent studies have also applied this methodology not only to analyze investor reaction to corporate events or news but also to other events such as pandemic outbreaks (e.g., SARS (Chen et al. 2007), COVID-19 (Pandey and Kumar 2021; Pandey and Kumari 2021; Wu et al. 2021)), airplane crashes (Gumanti et al. 2018), terrorist attacks (Gok et al. 2020; Hadi et al*.*, 2019), wars (Hudson and Urquhart 2015), climate change (Lee et al. 2015), and rumors (Chen and Kutan 2016). Investors aiming for unexpected positive returns on common stock investments frequently search for news and events that influence a company's stock price. More recently, new media sources and social networks have facilitated social interaction and spread expert opinions and sentiments, which may influence financial markets (Li et al. 2022; McGurk et al. 2020).

First, the study of events allows us to determine whether investors have reacted to corporate announcements or news in a manner that abnormal returns are generated. Second, if the event study concludes that the event has impacted the return on a given portfolio of assets, investors should consider the event to adjust the portfolio, especially if they expect that the event could reoccur.

Several tools are available for performing event studies. A non-systematic but intensive search for web access tools returned us with two paying options: the study tools by Schimmer et al. (2014)^1^ and the Wharton Research Data Services.^2^The former is a research application that focuses on scientific publications, whereas the latter covers teaching needs by adding a specific toolkit. We searched for additional tools by conducting the following search on Google Scholar: ("event studies" OR "event study") (simulator OR app OR web) (teaching OR learning). We found no additional academic references indicating the existence of an event study software for teaching purposes. We reviewed 100 articles of which nine were related to the application of event studies to teaching. Nevertheless, we found a spreadsheet to conduct an event study for teaching purposes in Reese and Robins (2017).^3^The authors provided an MS Excel-based tool using a market model and S&P 500 companies.

Eventstudytools is an online platform that performs event study computations where customers only need to upload the pertinent financial data, which they may obtain from any source, and parametrize their research. This platform has three research applications: abnormal returns, abnormal volumes, and abnormal volatility calculators. The platform allows several estimation models (market, market-adjusted, CAPM, comparison period mean-adjusted, Fama–French 3 factor, Fama–French momentum 4 factor, and Fama–French 5 factor models) and test statistics. Obtaining basic abnormal return results is free, but payment is needed for full access.

Wharton Research Data Services (WRDS) is a research platform for global institutions that provides services to authorized users. Among the analytical tools offered are “US Daily Event Studies,” “US Intraday Second-by-Second Event Studies,” “Long Run Event Studies,” and “International Event Studies.” Daily event studies are based on US data and are limited to four models: market, market-adjusted, Fama–French 3-factor, and Fama–French 3-factor plus momentum. Particularly relevant is WRDS's Classroom, a teaching toolkit created especially for faculty members introducing finance and business concepts in the classroom. The required data are uploaded directly from fee-based databases, which implies that not only an institutional but also a database subscription is needed to access the tools.

Our study fills research gaps in the domain by developing a set of MS Excel spreadsheets using the Fama–French five-factor model, which facilitates the in-class presentation, implementation, and analysis of abnormal returns and volumes. The set of MS Excel files could be used not only for teaching purposes but also for obtaining research results for a manual limited-size selection of companies. Furthermore, we offer a second tool that is especially relevant for research purposes, which helps overcome the limitations of spreadsheets. This tool is a free open-source R code and Shiny application that works by uploading data retrieved from both free and paid sources. This second tool allows for the computation and analysis of abnormal returns, volatility, and volume, adjusting on-demand for the event and estimation window length while controlling for confounding effects. The user can select among several return estimation models (market, Fama–French 3-factor, Carhart 4-factor, and Fama–French 5-factor) and test the significance of the abnormal results. No prior programming knowledge is required to use this tool. All these files, as well as the data needed to run the app, are available in the EventStudies4Finance repository^4^ (Serrano and Cuadros 2022). The event chosen to illustrate how both tools work is the announcement of the Pfizer and BioNTech vaccine on November 9th, 2020, and its impact on the thirty stocks that make up the Dow Jones Industrial Average (DJIA) Index.

The objectives of this study are twofold. First, it seeks to help instructors teach students to conduct event studies using the Fama–French five-factor model to obtain and analyze the expected rate of return. This study shows how to develop the necessary calculations step-by-step, offering not only results that can be traced but also calculation templates in MS Excel to facilitate the process. Second, we provide the financial community with a free tool to automatically calculate the expected rate of return coded using the R programming language, specifically the Shiny package, and compare it with traditional MS Excel-based methodologies. To the best of our knowledge, this is the first free tool of this type to be made available. The use of R is particularly relevant (Yan 2017) because a macro event that affects a large number of companies is being studied.

This study endeavors to contribute to education, research, and practice by providing both instructors and professionals with free ready-to-use tools (MS Excel spreadsheets, R code, and Shiny package) to undertake an event study. These tools, combined with the exercises presented, could be used by instructors to teach finance students how to perform event studies as well as the importance of mastering the use of large dataset gathering and analysis programmes in financial education (Fang and Zhang 2016). The same tools enable professionals to use the Fama and French (2015) model for any given market or portfolio of their choice. This is particularly relevant for this multivariant model, which requires large datasets that can only be processed efficiently with convenient applications such as those proposed in this exercise.

This study contributes to the literature by offering guidelines for the practical application of event studies and the Fama–French five-factor model, which is applied to obtain expected returns in MS Excel files made available to the community. We believe that these files could help students visualize how to apply the equations of this particular methodology and model.

The community is also equipped with a powerful tool to calculate abnormal returns, volatilities, and volumes using an R-dashboard. Although it is true that obtaining abnormal values using MS Excel helps visualize the process to be followed, when working with large samples, this calculation can be laborious. Moreover, mistakes can easily occur when calculations are performed manually. Therefore, this tool facilitates calculations for any sample size, without being prone to manipulation errors.

In the methodology section, the general steps to develop an event study analysis using the Fama–French five-factor model and generate MS Excel files are presented. This section also describes the classification of portfolios using a description file in R, as well as the usability of the R tool. Section three shows the main results of the study and discusses the two options presented. Finally, the conclusions are presented toward the end of this paper.

## Methodology

The impact of the announcement of the Pfizer and BioNTech COVID-19 vaccines on November 9, 2020, on the 30 companies that make up the DJIA index is considered in this study to illustrate the objective. This is a single-country example; specific methodological issues that arise when dealing with multiple countries can be found in Park’s (2004) study.

The event study methodology allows us to determine whether new information affects investors. Abnormal price changes are investors’ responses to information disclosure (Beaver 1968). If new information changes investors' valuation of a given stock, it will cause a significant variation in its price, which we refer to as an abnormal return (AR). This information is incorporated into a stock’s price almost as soon as it becomes public, assuming a semi-strong form of the efficient market hypothesis (Fama 1970). The underlying idea was to determine the existence of investor reactions. We establish an expected return and compare it to the actual return. If the difference between actual and expected returns is significant, we consider this difference to be an abnormal part of the investor's reaction.

Several models allow us to obtain an expected return, ranging from mean-adjusted, market-adjusted, and market models (Brown and Warner 1980; 1985; Dyckman et al. 1984) to more sophisticated models in which other factors are added to provide measures of risk adjustments. Armitage (1995) reviews the different models of expected returns and various approaches to measuring significance. The essential idea in multifactor models is that the expected return on an asset is a function of its systematic risk, as measured by a series of betas associated with the explanatory factors. Fama and French (1993) presented a three-factor model consisting of market risk, size, and value as sources of risk that determine expected returns. Market risk, already developed in the Capital Asset Pricing Model and Asset Pricing Model, is complemented here with microeconomic variables such as the size and relative value of the company to its book value. The size effect argues that stocks with small market capitalizations earn higher returns than those with large market capitalizations. The value effect suggests that the performance of stocks with low book prices is better than that of stocks with high book prices. Carhart (1997) published a four-factor model that builds on the Fama–French three-factor model. He added the momentum factor, which is created by subtracting the equal-weighted average of the highest-performing firms from the lowest-performing firms lagged by one month. The factor measures the tendency of a stock to continue moving in the direction in which it moved in the previous period.

Fama and French (2015) published the five-factor model presented below as Eq. (1), which adds two microeconomic risk factors to its multivariant expected return analysis. The model adds profitability (stocks with high operating profitability perform better) and investment factors (stocks of companies with high total asset growth have below-average returns) to estimate expected returns. Despite its limitations, this latter model better explains the expected returns on stock investments (Fama and French 2015; 2017; Huang 2019) among the ones considered. A comparison with actual data allowed us to identify abnormally positive and negative returns on the day of the event studied. Achieving abnormal positive returns and avoiding abnormal negative returns are common goals for investors and, consequently, are of interest to the financial community.

1 $$R_{i,t} - R_{ft} = \propto + \beta_{mt} \left( {R_{mt} - R_{f} } \right) + \beta_{SMB} SMB + \beta_{HML} HML + \beta_{RMW} RMW + \beta_{CMA} CMA + \in_{i,t}$$

Using the Fama–French five-factor model (Eq. (1)), the expected return (E(R_i,t_)) is obtained for each stock, after which the abnormal return (AR) is obtained as the difference between the actual return (R_i,t_) and expected return (E($${R}_{i,t})$$); thus, AR_i,t_ = R_i,t_-E(R_i,t_) is calculated. In the following sections, we describe how the calculations were performed in both MS Excel and R.

### Applied methodology framework using MS Excel

**Fig. 1:**
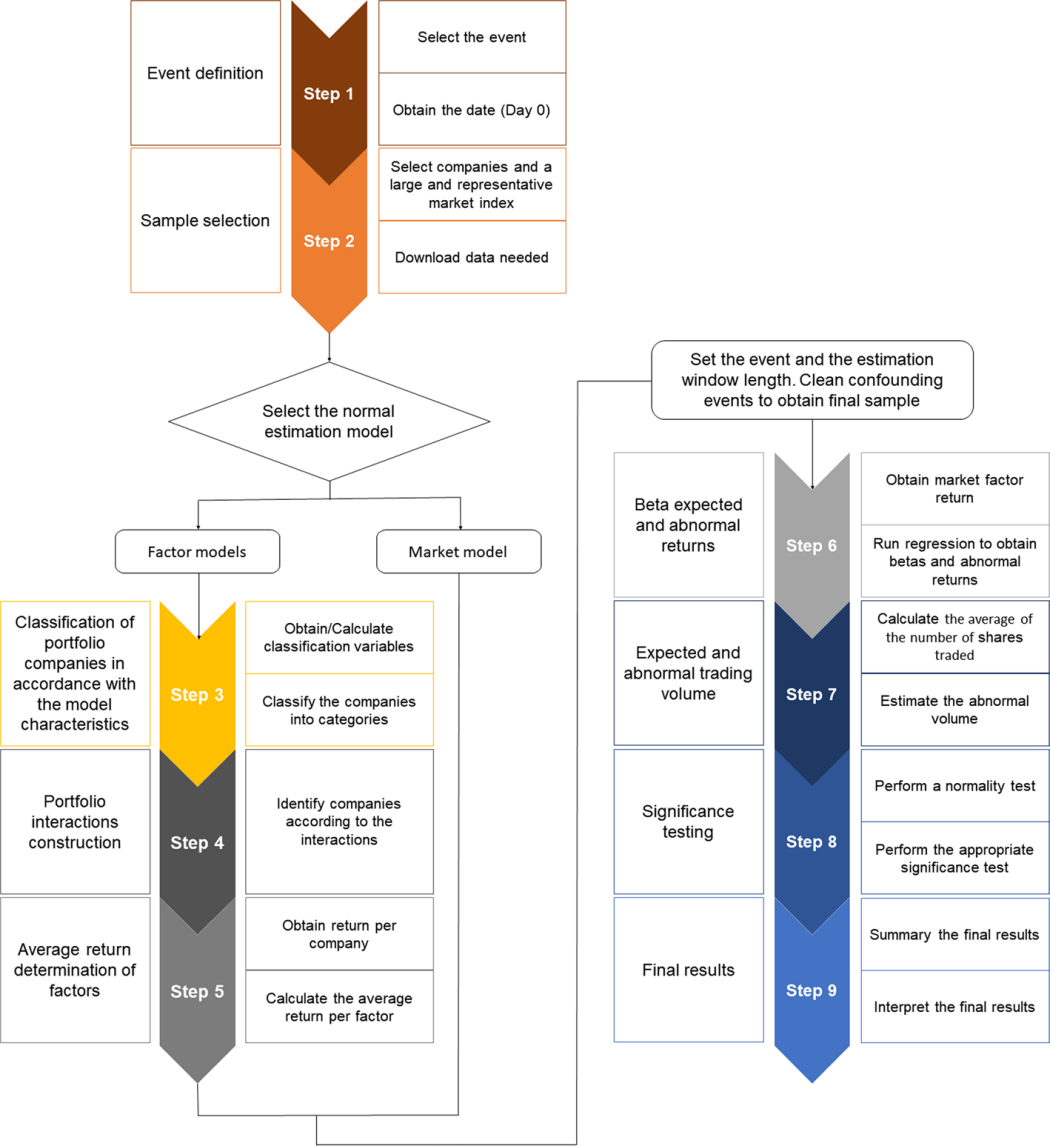
Flowchart of event study methodology. *Source*: Created by authors

To put the methodology into practice, Fig. 1 summarizes the nine steps to be undertaken, which are outlined below.

**Fig. 2:**
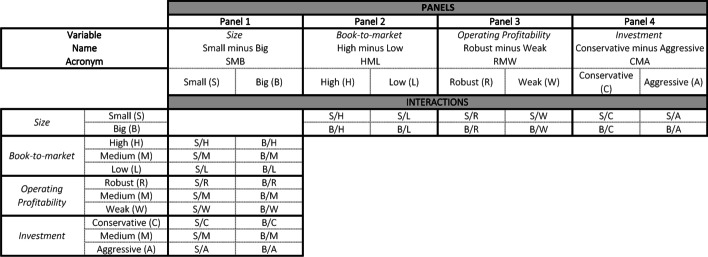
Summary of portfolios by patterns. *Source*: Created by authors
  We first assigned names to the range of cells containing small and big companies in MS Excel. This easy action simplifies the interaction determination: Select the cells to be named, click on the Name Box to the left of the formula bar, and type a one-word name on the list. We then copy-pasted the names of the companies classified earlier in the columns under the following characteristics: Small, High, Medium, Low, Robust, Medium, Weak, Conservative, Medium, and Aggressive to later use the VLOOKUP function. To make it easier to observe an interaction that is taking place, conditional formatting highlighted in green was added. For the example shown in Fig. 3, we searched for small and high companies to determine which companies would constitute the S/H portfolio. The formula returns the company name if it belongs to both groups or the #N/A error if it does not. Conditional formatting (green) was added when the output of the function differed from that of #N/A. By dragging the function down and sideways, a list of all the companies, which are simultaneously Small and High (S/H), Small and Medium (S/M), and Small and Low (S/L), could be obtained.

- *Step 1*: Identification of the event to be studied: The announcement on November 9, 2020, of the availability of an effective vaccine against COVID-19 was chosen as a significant event. In this study, the event date was t = 0. In this case, the date of the event was the same for all the companies studied. However, there may be other event studies (e.g., dividend or result announcements) in which the dates are different for the companies analyzed. When an event occurs after the financial market closes, the next day with available market information is taken as t = 0. This is especially relevant when selecting an event date for a multi-country event study.
- *Step 2*: Sample selection, market portfolio, and data: Companies comprising the sample are selected. In this exercise, there was one event per company, meaning that the number of companies and events were the same (n = 30) in the sample. The companies included in the DJIA index as of August 31, 2020 were chosen for the analysis. These 30 publicly owned companies are considered leaders in the U.S. economy. Additionally, the data for these companies are easily available, and the reduced number of companies allows step-by-step calculations to be performed in MS Excel.
  In addition, a market portfolio whose number of companies could be managed for manual calculation must be created. A point to be noted is that given the limitations of manual data computation in MS Excel, where companies have to be manually processed individually, we only considered the 30 stocks belonging to the DJIA index as a market portfolio for teaching purposes. However, for accurate factor returns, a more representative market portfolio with a much larger number of companies should be used to determine the value of reliable factors.
  It was assumed that all the data needed to perform the calculations were present. Therefore, it is essential to obtain accounting information for each company in the sample to construct portfolios. Annex A shows the values of the variables used in this study at t − 1 (December 31, 2019) and t − 2 (December 31, 2018). In addition, market information is needed, such as the daily closing price values of the companies in our sample, the market index (DJIA), and the return on a risk-free asset with short-term maturity (federal discount rate). Currently, free data sources are widely available (Yan 2017), although the data provided by S&P Capital IQ were used in this exercise.
- *Step 3*: Classify companies according to the requirements of the model’s characteristics: Small, Big, High, Low, Robust, Weak, Conservative and Aggressive companies.
  Small and Big: Small (S) and big (B) companies can be easily classified. Only one variable is required to work with market capitalization (*Market_Cap)*, which must be sorted from lowest to highest. Sorting was easily performed using the Excel SORT function. Fifty percent of the companies at the top of the list were small and the remaining were big. In accordance with Fama and French (1993), companies with negative equity were not considered for portfolio formation (in this exercise, Boeing Company, McDonald’s Corporation, and Home Depot, Inc.) for small and big groups, or for the next variable (High, Medium, or Low classification).
  High, Medium, and Low: To classify companies into one of the three categories, the book-to-market (B/M) ratio (Eq. (2)), sorted from the highest to the lowest, was used. Thirty percent of the companies at the top of the list were classified as high (H), 30% of the companies at the bottom of the list were classified as low (L), and the remaining 40% were classified as medium (M).
  2 $${\text{B}}/{\text{M}}\;{\text{ratio}} = \frac{{{\text{Equity}}_{{({\text{t}} - {1})}} }}{{{\text{Market}}\_{\text{Cap}}_{{({\text{t}} - {1})}} }}$$
  Robust, Medium, and Weak: To classify the companies into one of the three categories, the operating profitability ratio (Eq. (3)), sorted from the highest to the lowest, was used. Thirty percent of the companies at the top of the list were classified as Robust (R), 30% of the companies at the bottom were classified as Weak (W), and the remaining 40% in the middle as Medium.
  3 $${\text{Operating}}\;{\text{profitability}}\;{\text{ratio = }}\frac{{{\text{Revenue}}_{{({\text{t}} - {1})}} - {\text{COGS}}_{{({\text{t}} - {1})}} - {\text{ SG}}\& {\text{A}}_{{({\text{t}} - {1})}} - {\text{ Interest}}\_{\text{Expense}}_{{({\text{t}} - {1})}} }}{{{\text{Equity}}_{{({\text{t}} - {1})}} }}$$
  Conservative, Medium, and Aggressive: The investment ratio (Eq. (4)), sorted from the lowest to highest, was used to classify companies into one of these three categories. Thirty percent of the companies at the top of the list were classified as Conservative (C), 30% of the companies at the bottom were classified as Aggressive (A), and the remaining 40% in the middle as Medium.
  4 $${\text{Investment}}\;{\text{ratio}} = \frac{{{\text{Total}}\;{\text{Assets}}_{{({\text{t}} - {1})}} {-}{\text{ Total}}\;{\text{Assets}}_{{({\text{t}} - {2})}} }}{{{\text{Total}}\;{\text{Assets}}_{{({\text{t}} - {2})}} }}$$
  The first spreadsheet (File 1-Portfolios byhand.xlsx) shows how to classify companies belonging to the market portfolio in accordance with each of the patterns of the model (titled as step 3).
- *Step 4*: Portfolio interactions construction.
  Figure 2 shows the interactions among the company characteristics needed to build each portfolio. We identified the interactions between the patterns’ value, profitability, investment ratio, and company size using the VLOOKUP function in MS Excel. Accordingly, companies were identified in each portfolio. Using MS Excel is a feasible option for a relatively small number of companies. However, when the market portfolio to be used to develop factor returns includes a larger number of companies, it is more convenient to use alternative tools, such as the statistical package R, as will be shown later.

**Fig. 3:**
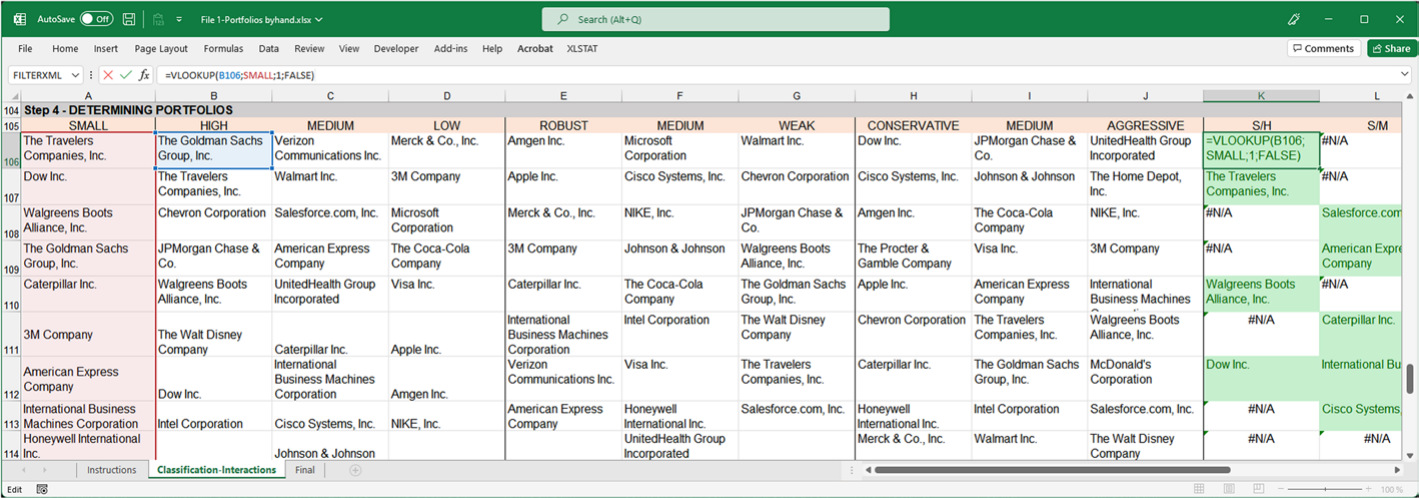
The VLOOKUP function for interaction determination. *Source*: Created by authors
  By repeating the process with the remaining columns and for big companies, companies belonging to the 18 portfolios listed in Fig. 2 (see Fig. 4 for the classification results) are obtained.

**Fig. 4:**
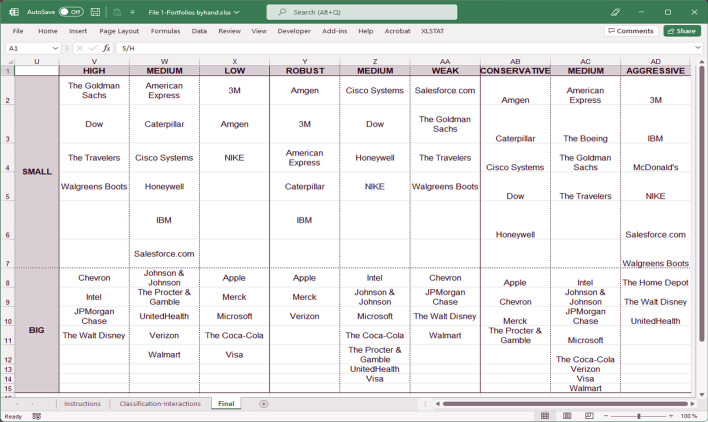
Companies for portfolio formation. *Source*: Created by authors
  Again, the first spreadsheet (File 1-Portfolios_Byhand.xlsx) shows how to obtain the companies which meet the conditions.
  Despite being able to use a simple spreadsheet to perform all of these calculations, when teaching the event study methodology, it is useful to make this study more automatic and reproducible. This is particularly true when a larger number of companies are used. Several approaches can be adopted in this regard. Because of its simplicity, the method consists of creating a spreadsheet template without the need to use VBA Macros. The MS Excel template developed in this study (second spreadsheet, File 2-Portfolios template.xlsx) allows users to obtain the companies' classification simply by entering the necessary data to perform the calculations (Fig. 5), as mentioned above.

**Fig. 5:**
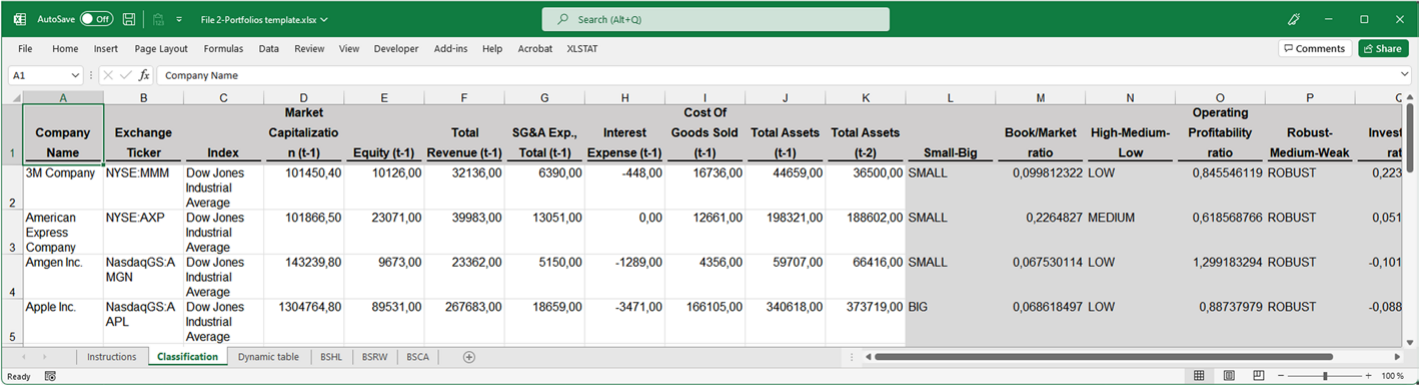
Automatic spreadsheet template. *Source*: Created by authors
  The data must be entered into the first tab, after which the classification is automatically obtained. However, the results of the second and optional tab might require a simple manual user modification because this tab contains a dynamic table that should be updated every time changes are made to the input data. This tab offers the user the possibility of interacting to obtain additional information from the data. However, as the complexity of the template increases, it becomes more difficult to work with a flat MS Excel file, and deriving the results automatically avoiding manual changes becomes complicated. Other approaches, such as programming languages, are useful in this context, as discussed later in this paper.
- *Step 5*: Average return determination of SMB, HML, RMW, and CMA.
  To determine the average return for a given portfolio, it is necessary to first obtain the return for each company. In this example, daily returns are obtained by applying Eq. (5) to adjust the closing daily prices downloaded from the Thomson Reuters database in MS Excel:
  5 $$R_{i,t} = ln\frac{{Closing\,price_{t} }}{{Closing\,price_{t - 1} }}$$
  The daily returns for each company were obtained between June 18, 2020 and November 11, 2020. The event study methodology requires a pre-event period, free of the event under analysis, to avoid confounding effects and to determine the coefficients to be used in the model. Some days within that period should remain unused for slope determination between the pre-event period and the window of days in which the effect of the information is analyzed. The adjusted stock price, which is the price of the stock after accounting for the effects of corporate actions, such as dividends and splits, was used.
  Once the returns for each company were calculated, the companies comprising each portfolio were selected and their average daily returns were calculated. Companies’ returns were copied and pasted into Excel AVERAGE (third spreadsheet, File 3-SMB HML RMW CMA Returns Determination byhand.xlsx). It is important to align all companies by date before using the AVERAGE function. The rows that contain the dates must match those of all companies.
  We now describe the variables and how companies choose to determine average daily returns.

#### Small minus big (SMB)

This variable considers the interaction of the size variable with the remaining variables in the model. Therefore, we must identify the companies belonging to the 18 different portfolios, which was executed based on the interactions between the classifications established in Fig. 2, Panel 1. This allowed us to determine the companies that must be used to calculate the average daily returns required for this variable. By forming portfolios that consider interactions for the calculation of the variables, we ensured that all explanatory variables were considered. The SMB daily returns are obtained by applying the following simple average:

6 $$SMB = 1/3\left( {SMBB/M \, + \, SMBProf \, + \, SMBInv} \right)$$

where

7 $$\begin{aligned} SMBB/M \, = & \, 1/3\left( {S/L \, + \, S/M \, + \, S/H} \right) \, - \, 1/3(B/L \, + \, B/M \, + \, B/H) \\ = & \; Simple \, average \, of \, returns \, for \, \left( {S/L} \right),\left( {S/M} \right) \, and \, \left( {S/H} \right) \\ & companies \, minus \, the \, simple \, average \, of \, big \, companies \, which \, are \, also \, low \, \left( {B/L} \right), \\ & medium \, \left( {B/M} \right), \, and \, high \, \left( {B/H} \right) \\ \end{aligned}$$

8 $$\begin{aligned} SMBProf \, = & \, 1/3\left( {S/W \, + \, S/M \, + \, S/R} \right) \, - \, 1/3(B/W \, + \, B/M \, + \, B/R) \\ = & \; Simple \, average \, of \, returns \, for \, \left( {S/W} \right), \, \left( {S/M} \right) \, and \, \left( {S/R} \right) \\ & companies \, minus \, the \, simple \, average \, of \, big \, companies \, which \, are \, also \, weak \, \left( {B/W} \right), \\ & medium \, \left( {B/M} \right), \, and \, robust \, \left( {B/R} \right) \\ \end{aligned}$$

9 $$\begin{aligned} SMBInv = & 1/3(S/A \, + \, S/M \, + \, S/C) \, - \, 1/3\left( {B/A \, + \, B/M \, + \, B/C} \right) \\ = & \; Simple \, average \, of \, returns \, for \, \left( {S/A} \right), \, \left( {S/M} \right) \, and \, \left( {S/C} \right) \, companies \, minus \, the \, simple \, average \\ & of \, big \, companies \, which \, are \, also \, aggressive \, \left( {B/A} \right), \, medium \, \left( {B/M} \right), \, and \, conservative \, \left( {B/C} \right) \\ \end{aligned}$$

Once (7)–(9) have been obtained; they must be averaged to obtain the daily values for (6) or SMB in return.

#### High minus low (HML)

This corresponds to the difference in stock returns between the average returns on a portfolio of securities made up of companies with a high B/M ratio and the average return of a portfolio made up of companies with a low B/M. We also consider its interaction with size (Fig. 2, Panel 2) and obtain the HML daily returns by applying the following simple average:

10 $$\begin{aligned} HML \, = & \, 1/2\left( {S/H \, + \, B/H} \right) \, - \, 1/2\left( {S/L \, + \, B/L} \right) \\ & = \; Simple \, average \, return \, for \, high \, B/M \, ratio \, companies \, which \\ \, & are \, also \, small \, \left( {S/H} \right) \, and \, big \, \left( {B/H} \right) \, minus \, the \, simple \\ & average \, of \, low \, companies \, which \, are \, also \, small \, \left( {S/L} \right) \, and \, big \, \left( {B/L} \right) \\ \end{aligned}$$

#### Robust minus weak (RMW)

corresponds to the difference in stock returns between the average return of a portfolio of securities made up of companies with high profitability and the average return of a portfolio made up of companies with low profitability. We also consider its interaction with size (Fig. 2, Panel 3) and obtain the RMW daily returns by applying the following simple average:

11 $$\begin{aligned} RMW \, = & \, 1/2\left( {S/R \, + \, B/R} \right) \, - \, 1/2\left( {S/W \, + \, B/W} \right) \\ = & \; Simple \, average \, return \, for \, robust \, companies \, which \, are \, also \, small \, \left( {S/R} \right) \\ & and \, big \, \left( {B/R} \right) \, minus \, the \, simple \, average \, of \, weak \, companies \\ & which \, are \, also \, small \, \left( {S/W} \right) \, and \, big \, \left( {B/W} \right) \\ \end{aligned}$$

#### Conservative minus aggressive (CMA)

This corresponds to the difference in stock returns between the average return of a portfolio of securities comprising companies with a low investment ratio and the average return of a portfolio comprising companies with a high ratio. We also consider its interaction with size (Fig. 2, Panel 4) and obtain the CMA daily returns by applying the following simple average:

12 $$\begin{aligned} CMA \, = & \, 1/2\left( {S/C \, + \, B/C} \right) \, - \, 1/2\left( {S/A \, + \, B/A} \right) \\ = & \, Simple \, average \, return \, for \, conservative \, companies \, which \, are \, also \, small \, \left( {S/C} \right) \\ & and \, big \, \left( {B/C} \right) \, minus \, the \, simple \, average \, of \, aggressive \, companies \, which \\ & are \, also \, small \, \left( {S/A} \right) \, and \, big \, \left( {B/A} \right) \\ \end{aligned}$$

The results in File 3 are based on the 30 stocks belonging to the DJIA. As stated before, we selected the market portfolio for developing factor returns because its limited number of components allowed us to apply the methodology manually to meet our teaching objectives.

Nevertheless, we acknowledge that a factor-return estimation based on a limited number of companies can return misleading factor values. As an alternative, daily data for the variables Rm-Rf, SMB, HML, RMW, and CMA can be obtained from Kenneth R. French's website (https://mba.tuck. dartmouth. edu/pages/faculty/ken.french/data_library.html). The website allows users to download ready-to-use variable factors calculated using all the companies listed on the New York Stock Exchange (NYSE), American Stock Exchange (AMEX), and National Association of Securities Dealers Automated Quotations (NASDAQ). Consequently, we believe that estimating the factors and abnormal returns using Kenneth R. French’s website variable returns could be a more appropriate methodology for obtaining accurate factors for research purposes.

- *Step 6*: Factor values and expected and abnormal returns.

Once the daily values of the SMB, HML, RMW, and CMA are obtained, the value of the market factor (R_m_-R_f_), the last variable needed for the estimation, must be calculated. This daily value is the difference between the daily return on the DJIA (R_m_) and that of a risk-free asset (R_f_). In this exercise, R_f_ was obtained using the yearly discount rate set by the Federal Reserve during the period studied, assuming a 360-day year. The equation used to obtain daily returns based on the annual discount rate is as follows:

13 $$R_{f} = \left( {1 + discount\,rate} \right)^{\frac{1}{360}} ) - 1$$

As the discount rate set by the Federal Reserve remained at 0.25% during the period studied, R_f_ remained constant during that period.

To obtain R_m_, the procedure is the same as that for Eq. (5), but the closing values for the index must be selected:

14 $$R_{m} = {\text{ln}} \frac{{Closing\,Value\,DJIA_{t} }}{{Closing\,Value\,DJIA_{t - 1} }}$$

Once both R_m_ and R_f_ are obtained, the market factor (R_m_-R_f_) is calculated to apply the model as the difference between the variables.

An MS Excel file can now be set up with all the data, and a regression can be run to find the factor values that can be used to forecast expected returns. As stated in Step 5, the daily values of (R_m_-R_f_), SMB, HML, RMW, and CMA can also be obtained by accessing Kenneth R. French's website.

Before running the regression, the length of the event window and estimation period must be set because both determine the number of variable values to be used in the estimation. The conventional analysis in an event study calculates the magnitude of the abnormal return (AR) not only on the event day but also for the pre-event and event windows. As stated in Mackinlay (1997, p. 14): “It is customary to define the event window to be larger than the specific period of interest. This permits examination of periods surrounding the event.” The author urges including at least the day of the announcement and the day after to capture the price effects when announcements occur after the stock market closes on the announcement day. Moreover, if there is leakage of information before the announcement, we should observe a market reaction before the announcement date; however, there may also be a market reaction after the information is disclosed (Ball and Brown 1968; Ball and Kothari 1991; Sorescu 2017). Therefore, to study these possible effects, the period [− 2, + 2] was used as the event window.

Once the length of the event window is set, the estimation window must be defined; the usual choice is to use the period prior to the event window (Mackinley 1997). Because the estimation period must be free of event influence to determine the normal performance of the stock, this estimation window cannot include the event period; unused days between the event and the estimation window should also be excluded.

**Fig. 6:**
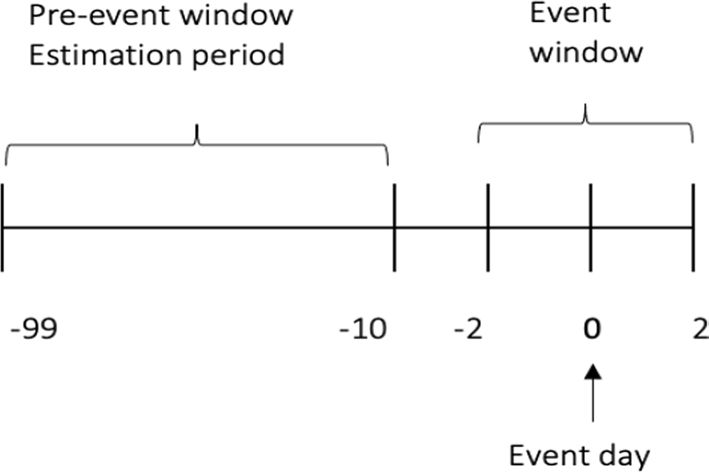
Returns pre-event and event window. *Source*: Created by authors

**Fig. 7:**
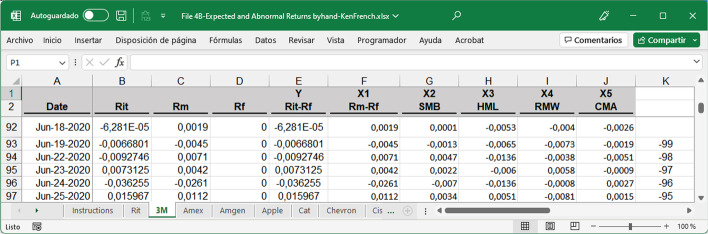
Partial 3 M spreadsheet structure for running a regression. *Source*: Created by authors

The values used for the regression are the returns in the 90 trading days prior to the event for each security. The returns of the securities studied between day t = − 10 (October 26, 2020) and t = − 99 (June 19, 2020) are used (see Fig. 6). The output was a spreadsheet, as shown in Fig. 7, for each of the 30 companies studied.

To perform the analysis, an Excel Analysis ToolPack add-in was installed on the computer. Multivariate regression analysis must be performed for each security to calculate the factor values for Eq. (1). To run regressions in MS Excel, the following need to be selected: Data, Data Analysis, Regression, and then follow the menu, where R_i,t_ is the dependent variable to be explained by the five-factor model (Fig. 7). The intercept was set to 0.

**Fig. 8:**
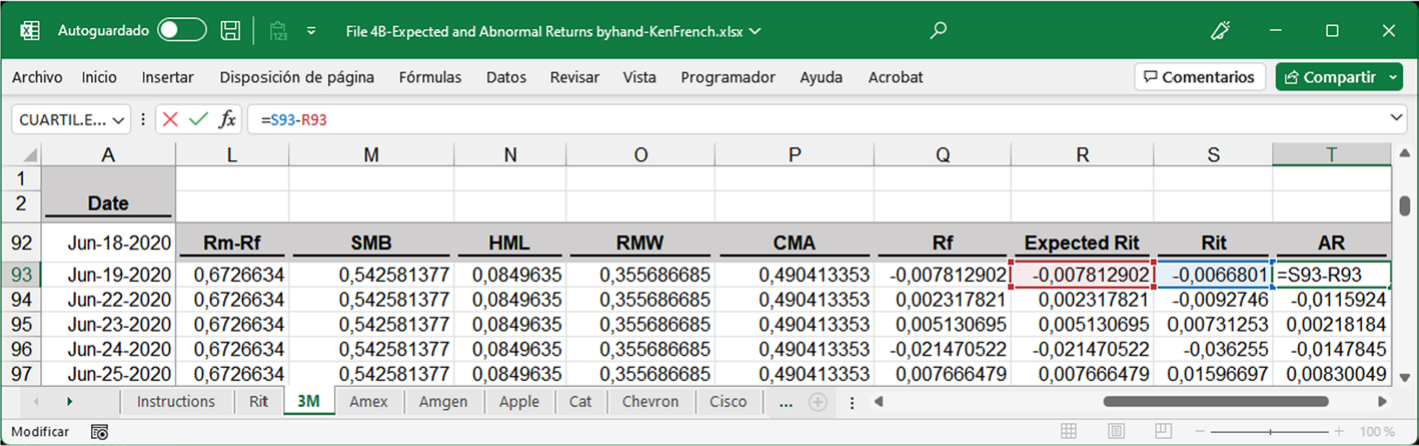
Partial 3 M spreadsheet structure to calculate expected and abnormal returns. *Source*: Created by authors

The summary output obtained after running each regression will return, among other information, the five coefficients needed to calculate the expected return. A regression per company must be run to obtain five coefficients for each of the 30 securities studied. Once the coefficients have been obtained, the expected daily return for the pre-event window, event window, and for each security can be calculated by applying Eq. (1). This information can be found in the fourth spreadsheet. File 4A-Expected and Abnormal Returns byhand-DJIA.xlsx shows the results based on the variable values manually obtained in File 3. File 4B-Expected Abnormal Returns byhand-KenFrench.xlsx presents the results based on Kenneth R. French's website variables. Notably, the dependent variable of the regression represents the daily return of each security (R_i,t_) minus the daily risk-free rate (R_f_). The AR per day was obtained by applying Eq. (15) (Fig. 8).

15 $$AR_{i,t} = R_{it} - E_{{\left( {Ri,t} \right)}}$$

- *Step 7*: Expected and Abnormal trading volume.

As stated in Kim and Verrecchia’s (1991, p. 303) study, “[…] price change, as Beaver (1968) points out, reflects the average change in traders’ beliefs due to the announcement, whereas trading volume reflects trader’s idiosyncratic reactions. […] volume reflects the sum of differences in traders’ reaction; the change in price measures only the average reaction.” Therefore, analyzing abnormal trading volumes can complement the picture of abnormal prices regarding investors’ reactions to a given announcement. If investors’ reactions to an event given by abnormal prices are positive (negative) and the abnormal volume is higher (lower) than normal, we can conclude that the force behind the event impact is strong (weak).

The expected trading volume is easier to calculate than the returns because only the average number of shares traded is calculated. We obtain the abnormal trading volume by comparing the expected volume with the daily volume. As for returns, we need a measure of non-event-related (or normal) volume to analyze abnormal trading activity. Our choice of benchmark to establish a normal trading volume follows the average volume model (Campbell et al. 1997), considering not only the pre-event days but also the post-event days (Womack 1996). Therefore, we define abnormal trading volume (AV) for stock *i* on day *t* as:

16 $$AV_{i,t} = \frac{{V_{i,t} }}{{\left( {\mathop \sum \nolimits_{t = - 55 }^{ - 11} V_{i,t} + \mathop \sum \nolimits_{t = 11 }^{55} V_{i,t} } \right) { \times } \frac{1}{90}}}$$

where: $V_{i,t}$ is the traded volume of stock and we consider not only the pre-event period [− 55, − 11] but also a post-event period of the same size [11, 55].

**Fig. 9:**
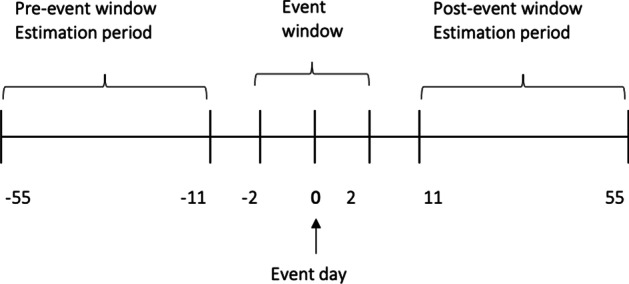
Volume pre-event and event window. *Source*: Created by authors

As shown in Eq. (16), the average volume measured by the number of shares traded 90, 45, and 45 days after the event is first calculated. The period between t = − 11 and t =  + 11 is not taken into consideration (see Fig. 9). As with AR, we not only analyzed the event day but also set an event window.

The AVERAGE function in Microsoft Excel can be used to obtain expected trading volumes. The fifth spreadsheet (File 5-Expected and Abnormal Volume 3 M byhand.xlsx) shows how to perform this analysis for 3 M.

- *Step 8*: Abnormal returns and volume analysis; testing significance.

After completing Steps 6 and 7, we obtained the daily abnormal returns and volumes per company. They are always different from zero because the actual and expected returns and volumes are not the same. In an event study, it is common to obtain the aggregate market reaction by averaging the abnormal returns of all events. Once the abnormal returns of all securities have been determined, per event, we average them daily (Eq. (17)), starting on day − 99 and finishing on day + 2, to obtain the daily average abnormal return (AAR).

17 $$AAR_{t} = \frac{1}{N} \mathop \sum \limits_{i = 1}^{N} AR_{i,t}$$

We can determine the aggregate market reaction to announcements by examining the AAR on day t = 0: The results may be negative or positive. If the abnormal return is positive (negative), it means that investors' decisions regarding the announcement have been evaluated positively (negatively) and that they have therefore decided to buy (sell).

Positive and negative AR cancel each other out unless most investors have either positive or negative reactions to the event. When investors cancel each other by taking opposite trading positions, abnormal price changes are not observed. Therefore, we also examine stock price volatility around the event date, calculated as the absolute value of abnormal returns (AbsAR), to avoid compensating effects.

We then proceed in the same manner as with AR, but adjust the abnormal value by subtracting the mean value of the pre-event period (Eq. (18)). This allows us to obtain the absolute value of average abnormal return per day (AbsAAR) and determine whether the stock shows controversy among investors (volatility increases) or not (not significant or decreases) with the event.

18 $$AbsAAR_{t} = \frac{1}{N} \mathop \sum \limits_{i = 1}^{N} \left| {AR_{i,t} } \right| - \overline{{AbsAR_{t} }}$$

where $\overline{{AbsAR_{t} }}$ is the average of AbsAR_t_ for the pre-event period.

As with the returns, once abnormal daily volumes have been computed for each firm, the average abnormal trading volume (AAV) on day t is calculated as:

19 $$AAV_{t} = \frac{1}{N} \mathop \sum \limits_{i = 1}^{N} AV_{i,t}$$

To respond to the question related to how the stock market reacted to the announcement (day 0) of an effective vaccine, an answer has to be provided to the null hypothesis. The null hypotheses to be tested are established according to the methodology of event studies (Brown and Warner 1985) in its null version: Hypothesis 1, H01: The announcement of an event does not produce statistically significant abnormal returns compared to an ordinary day. Hypothesis 2, H02: The announcement of an event does not produce statistically significant abnormal absolute returns compared to an ordinary day. Hypothesis 3, H03: The announcement of an event does not produce an abnormal volume that is statistically significant compared with an ordinary day.

The assumption that stock returns are normally distributed is common in theoretical financing. This is because of the assumption that stock returns should be equally distributed and independent if stock prices are random. If sufficient data are collected, the central limit theorem can be applied. However, there is evidence of non-normality in the daily return distribution (Corrado 1989). In addition, there is evidence that in event-date clustered analyses of returns, test statistics cannot assume the independence of AR, and non-parametric tests have proven to be robust against event-induced volatility and cross-correlation (Kolary and Pynnönen 2010).

Testing for the significance of AARs requires testing the normal distribution of AAR, AbsAAR, and AAV. In this exercise, we propose the Kolmogorov–Smirnov normality test. The statistical significances of AAR, AbsAAR, and AAV were compared using a t-test when the data were normally distributed and independent of abnormal results. Equations (20)-(22) test the significance of abnormal returns, volatility, and volume, respectively.

20 $$t - student \,\,AAR_{i,t} = \frac{{AAR_{t} }}{{S_{AAR} }}$$

21 $$t - student \,\,AbsAAR_{i,t} = \frac{{AbsAAR_{t} }}{{S_{AbsAAR} }}$$

22 $$t - student \,\,AAV_{i,t} = \frac{{AAV_{t} }}{{S_{AAV} }}$$

where $S_{AAR}$, $S_{AbsAAR}$, and ${AAV}$ are the standard deviations of the estimation periods for AARs, AbsAARs, and AAVs, respectively.

For non-normally distributed results and/or research frameworks when the independent distribution of abnormal results cannot be assumed, we propose the non-parametric Corrado (1989) rank test, in which a company's AR, AbsAR, and AV in the whole series are transformed into ranks (K_i,t_) over the combined period, including the estimate and event window (T_i_). The test for abnormal returns is as follows:

First denote the rank of the AR_i,t_ over the combined period:

23 $$K_{i,t} = rank \left( {AR_{i,t} } \right)$$

Under the null hypothesis of no event impact, AR should be an arbitrary random value, and consequently, an arbitrary rank position. The test compares the ranks in the event period with the expected average rank under the null hypothesis of AR absence. The statistic for the null hypothesis is

24 $$statistic = \frac{{\frac{1}{N }\mathop \sum \nolimits_{i = 1}^{N} (K_{i,0} - \overline{{K_{i} )}} }}{{S(\overline{K)} }}$$

where:

25 $$S\left( {\overline{K}} \right) = \sqrt{\frac{1}{T}} \mathop \sum \limits_{t = 1}^{T} \left( {\frac{1}{{N^{2} }}\mathop \sum \limits_{i = 1}^{N} \left( {K_{i,t } - \overline{{K_{i} }} } \right)} \right)^{2}$$

The most common method for analyzing performance over longer time intervals is to use cumulative abnormal returns, which aggregate the abnormal returns for different periods. The cumulative value of the average abnormal returns (CAAR and CAbsAR) was calculated by aggregating the daily abnormal returns for several time intervals (a,b) within the event window [− 2, 2]. Performance not only in the event window but also over longer periods before and after the event may require examination.

26 $$CAAR = \mathop \sum \limits_{t = a}^{b} AAR_{t}$$

27 $$CAbsAAR = \mathop \sum \limits_{t = a}^{b} AbsAAR_{t}$$

This procedure allows us to determine market reactions that require more than one day to appear. One common reason that may require a two-day cumulative abnormal return is that the date on which the event took place cannot be determined exactly.

The cumulative average abnormal volume (CAAV) was obtained by adding the average daily abnormal volume across different time intervals (*a, b*) within the event window [− 2, + 2].

28 $$CAAV = \mathop \sum \limits_{t = a}^{b} AAV_{t}$$

We test the significance of the event firms’ cumulative average abnormal performance (CAAR, CAbsAAR, and CAAV) over a longer event period within the event window. As before, the null hypothesis is that the expected cumulative price and volume changes over a period are zero. This hypothesis can be tested in a similar way to testing a one-day AAR, and the statistics are defined as follows:

29 $$statistic\,CAAR = \frac{CAAR}{{\sqrt {N } S_{AAR} }}$$

30 $$statistic\,CAbsAAR = \frac{CAbsAAR}{{\sqrt {N } S_{AbsAAR} }}$$

31 $$statistic\,CAAV = \frac{CAAV}{{\sqrt {N } S_{AAV} }}$$

where* N* is equal to the number of aggregated days across the time interval.

To test for cumulative values under the non-parametric test, we proceed as in Eqs. (29)-(31); however, under this hypothesis, we accumulate the rank positions within the event window. Additionally, the standard deviations must be changed by the value of (25).

You may also consider applying the test proposed in Ataullah et al. (2011), which considerably simplifies the computational procedures of the Corrado (1989) test. The modified Corrado test is an interesting alternative to existing tests since it has high efficiency and few distributional assumptions.

To that end, we need to check whether AAR, AbsAAR, and AAV in the event window are statistically different (significant) from those on an ordinary day. Two different templates that only required inserting the AR and AV of individual companies by copying-pasting from previous individual files were provided to easily complete this step. The first template allows us to obtain the AAR and CAAR and perform a normality test, significance analysis using the t-test, significance analysis using the Corrado test, and AbsAAR and CAbsAAR to estimate the volatility of returns, where normality, t-, and Corrado tests are also performed (File 6-Abnormal Returns Analysis template.xlsx). The use of this template was illustrated by copy-pasting the AR for each company obtained from File 4B. Therefore, Steps 8 and 9 are based on the factor returns downloaded from French's website. Additionally, at the end of Step 9, we compare these results with those obtained using the factor returns calculated from companies listed on the DJIA (File 4A).

In the second template (File 7-Abnormal Volume Analysis template.xlsx), the same analysis is performed for volume, inserting an abnormal volume of individual corporate files and considering the pre- and post-event periods.

As previously stated, if the difference between the magnitude of abnormal values is high or low enough to test significantly different from that of an ordinary day compared to the pre-event period, it is concluded that the announcement affected the firm value and volume traded.

**Fig. 10:**
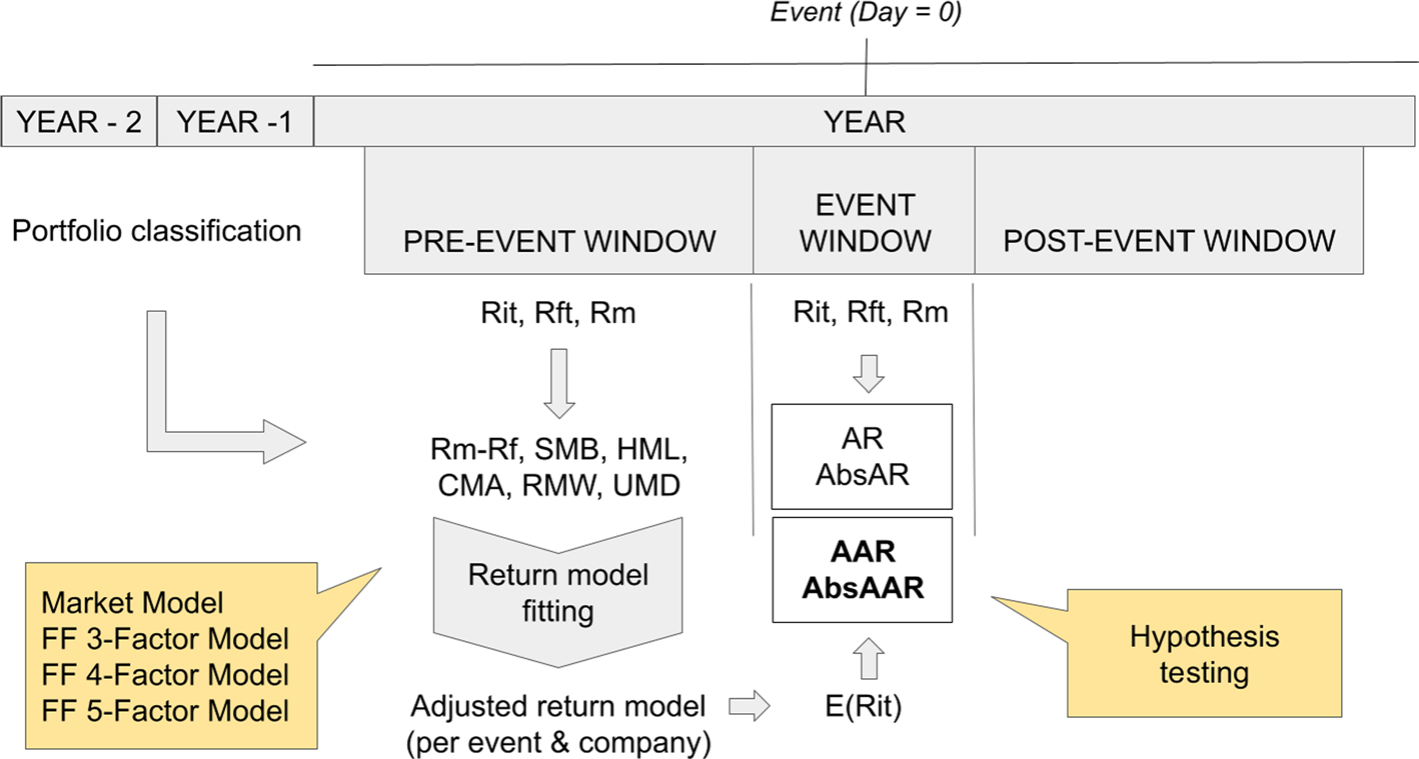
Summary of the process of testing for significant average abnormal returns and average abnormal volatilities. *Source*: Created by authors

**Fig. 11:**
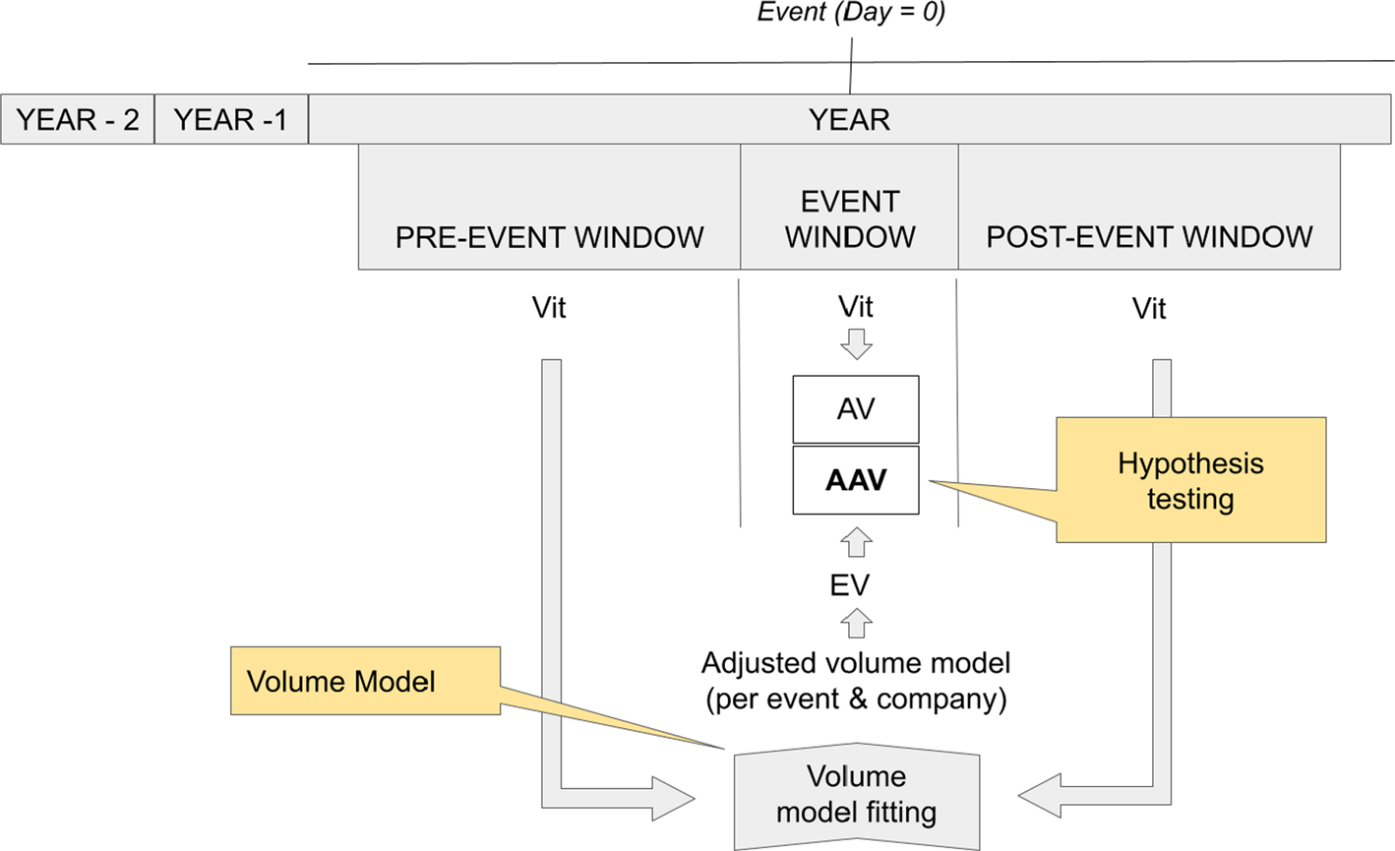
Summary of the process of testing for significant average abnormal volumes. *Source*: Created by authors

The research framework of this study is summarized in Fig. 10 for the determination of average abnormal returns and volatilities and in Fig. 11 for average abnormal volumes. These figures allow us to visualize the steps taken to test the hypotheses in an event study.

- *Step 9*: Showing the results.

Event studies are usually presented in tables that include six datasets: AAR, AbsAAR, AAV, CAAR, CAbsAAR, and CAAV. The statistical value for each is also shown and calculated using either the t-test or the Corrado test. The statistical value depends on whether the values follow a normal distribution and whether the independence of AR can or cannot be assumed. In this respect, we use non-parametric statistics to illustrate the results because we understand that we cannot assume the independence of AR (Kolary and Pynnönen 2010).

For instance, Table 1 shows the results obtained after processing the AR and AV using the sixth spreadsheet (File 6-Abnormal Returns Analysis template.xlsx) and the seventh spreadsheet (File 7-Abnormal Volume Analysis template). Following the results, the announcement of the vaccine had no relevant AAR or CAAR from t = − 2 to t = 0. However, when studying AbsAAR and CAbsAAR as proxies for market volatility, the statistical results of the Corrado test were relevant at t = 0. Therefore, it can be concluded that the AAR and CAAR are not sufficient for understanding the impact of the event studied. Possible compensating effects exist between the AAR of different stocks with positive and negative impacts on returns because the event studied needs to be considered, as shown in Annex B. The same conclusion is reached when examining Panel 3 of Table 1, which shows the AAV and CAAV, where the statistical results of the Corrado test are relevant. The AAV is relevant for the event day, whereas the CAAV is relevant for the intervals [0,1] and [0,2]. This means that the volume traded on the day of the event and the following day was abnormally higher than average.

**Table 1:** This table summarizes the AAR, CAAR, AbsAAR, CAbsAAR, AAV and CAAV on and around the date of the announcement of Pfizer’s COVID-19 vaccine for the companies listed on the DJIA index. Results obtained using Kenneth R. French factor returns

Event Day	N = 30		N = 30		N = 30	
	Panel 1		Panel 2		Panel 3	
	AAR (%)	Corrado	AbsAAR (%)	Corrado	AAV (%)	Corrado
− 2	− 0.053	− 0.0696	0.145	0.7698	− 2.90	0.1427
− 1	0.051	0.5567	− 0.128	− 0.2063	− 22.80	− 1.1558
0	1.330	1.4653	1.990	3.5355***	93.50	2.3924**
1	0.665	2.0181**	0.418	1.6309	34.90	1.6322
2	− 0.503	− 1.1521	0.160	1.0238	− 8.40	− 0.2597

Period	N = 30		N = 30		N = 30	
	Panel 1		Panel 2		Panel 3	
	CAAR (%)	Corrado	CAbsAAR (%)	Corrado	CAAV (%)	Corrado
[− 2,0]	1.328	1.1272	2.007	2.3666**	67.60	0.7964
[− 1,0]	1.381	1.4297	1.862	2.3541**	70.60	0.8744
[0,1]	1.995	2.4631**	2.408	3.6532***	128.40	2.8459***
[0,2]	1.491	1.3460	2.568	3.5739***	119.90	2.1737**

Source: Created by authors

Asterisks indicate significance at the 5% (**) and 1% (***) levels

Graphs are widely used to depict the results of event studies. For instance, in this study, CAAR (Graph 1) and CAAV (Graph 2) are shown from days t = − 10 to + 10 of the vaccine announcement, but the cumulated period can be increased to analyze a longer period. Abnormal returns on day t = − 30 can be accumulated to determine whether there was a run-up and can be extended until day t =  + 30 to determine the duration of the announcement effect.

In this example, the use of a graph allows us to observe that the cumulative impact is relevant from day t = − 1 to + 1, which reinforces the impact of the event on the stocks studied.
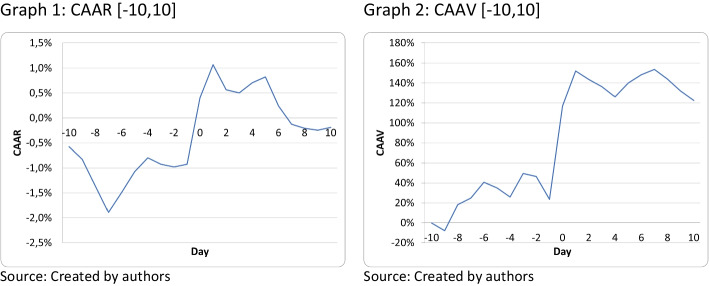


Finally, to complete the analysis, we present a comparison between the AAR and AbsAAR results under the factor returns obtained using the DJIA companies (Table 2, panels 1 and 2) and the Kenneth R. French website factor returns (Table 1, panels 1 and 2). Panel 3 was not compared because the traded volumes do not depend on the model used to calculate abnormal returns.

**Table 2:** This table summarizes the AAR, CAAR, AbsAAR, CAbsAAR, AAV and CAAV on and around the date of the announcement of Pfizer’s COVID-19 vaccine for the companies listed on the DJIA index. Results obtained using factor returns from DJIA

Event Day	N = 30		N = 30		N = 30	
	Panel 1		Panel 2		Panel 3	
	AAR (%)	Corrado	AbsAAR (%)	Corrado	AAV (%)	Corrado
-2	− 0.060	− 0.2674	0.073	0.7734	− 2.90	0.1427
-1	0.050	0.5784	− 0.147	− 0.3867	− 22.80	− 1.1558
0	0.317	− 0.5037	1.901	3.9270***	93.50	2.3924**
1	0.199	1.5175	0.590	1.9894**	34.90	1.6322
2	0.001	0.4664	0.016	− 0.0837	− 8.40	− 0.2597

Period	N = 30		N = 30		N = 30	
	Panel 1		Panel 2		Panel 3	
	CAAR (%)	Corrado	CAbsAAR (%)	Corrado	CAAV (%)	Corrado
[− 2,0]	0.307	− 0.1113	1.828	2.4906**	67.60	0.7964
[− 1,0]	0.367	0.0528	1.754	2.5034**	70.60	0.8744
[0,1]	0.515	0.7168	2.491	4.1836***	128.40	2.8459***
[0,2]	0.517	0.8546	2.507	3.3676***	119.90	2.1737**

Source: Created by authors

Asterisks indicate significance at the 5% (**) and 1% (***) levels

Referring to Panels 1 and 2, we observe that AAR and AbsAAR have different values although they follow similar trends. We have highlighted three small differences in significance. First, on Day 1, there is no significant AAR when using DJIA companies (t = 1.5175); however, there is a significant and positive AAR when using the Kenneth R. French factors (t = 2.0181). Second, AbsAAR on Day 1 appears to be significant using the DJIA companies (t = 1.9894), whereas when using the Kenneth R. French factors, AbsAAR does not appear to show distinct behavior from an ordinary day (t = 1.6309). Third, when we consider the accumulated period [0,1], we find differences between the two estimates: using DJIA, CAAR is not statistically significant (t = 0.7168), while using French factors, the Corrado test shows a statistically significant value of t = 2.4631 for the same period.

In addition to these differences, the major conclusions of the event study were similar in both cases. There was a significant difference in AbsAAR on Day 0, and CAbsAAR was significantly different from the average in all the periods considered.

### Methodology framework applied using R

In the previous section, we presented a methodology applied to MS Excel. However, as mentioned earlier, the larger the sample size, the more difficult it is to efficiently use MS Excel. Therefore, this section presents the use of programming languages as an alternative to MS Excel when teaching how to undertake event studies. Because most finance students may not have programming skills, a dashboard was created using Shiny, an open-source R package. The dashboard allows users to obtain results and interact with the data without coding. It is programmed to estimate the factors and abnormal returns in exactly the same way as explained with MS Excel. Indeed, the user would be able to select between preparing their own variable returns based on the selection of companies loaded into the program (sample data) or using the variable values published by Kenneth R. French.

**Fig. 12:**
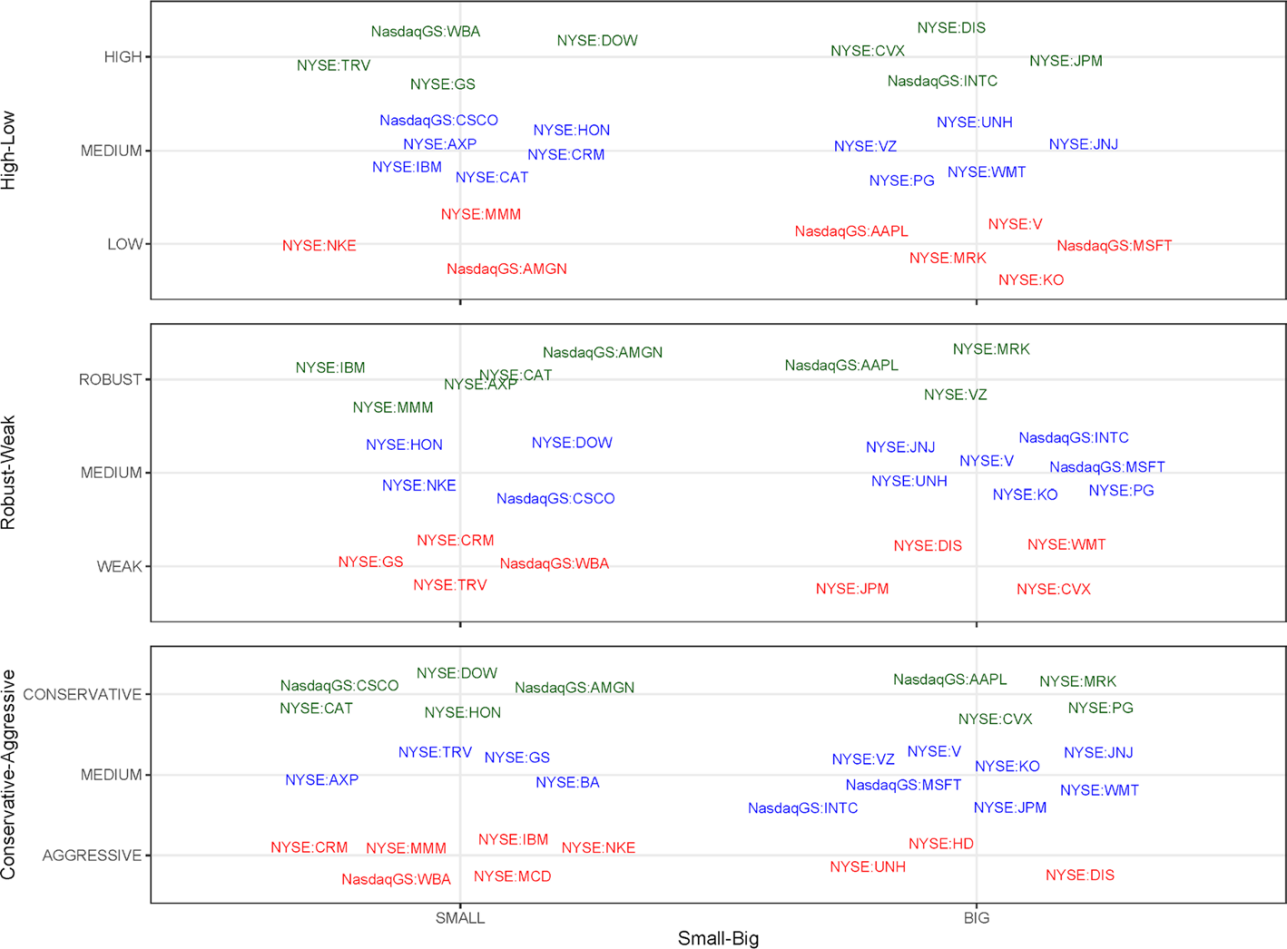
Portfolio classification using R. *Source*: Created by authors

The programming environment used in this exercise was R, one of the most widely used open-source programming languages for data analytics, and RStudio, an integrated development environment for R. RStudio includes an editor window that supports direct code execution, facilitating the reproducibility of the analysis. As shown in Fig. 12, a graphical classification was obtained using the R package ggplot2. It can be created by sourcing the script PortfoliosClassification.R in R.

**Fig. 13:**
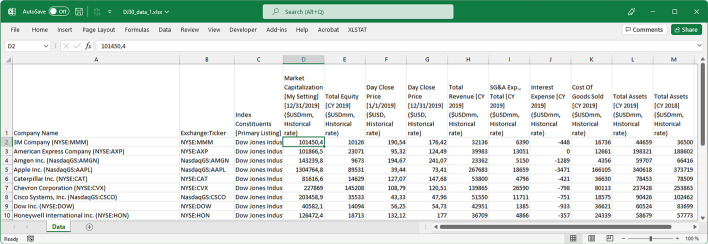
Sample of an MS Excel file with the data required for the portfolio classification. *Source*: Created by authors

The data required to run this snippet were an MS Excel file (DJ30_data_1. xlsx) with the data for the stock sample downloaded from S&P Capital IQ (Fig. 13).

As mentioned earlier, a dashboard that allows users to obtain results, similar to MS Excel, was created using Shiny. Five R files—ui.R, server.R, global.R, functionsMarketVolumes.R, and functionsAbnormalReturns. R—were generated to develop a dashboard. The ui file, which controls the layout and appearance of the app, and the server file, which contains the instructions for building the application, are mandatory for the dashboard, although a single file called the app could have been created instead. Both files must be in the same folder that contains a single ui and a single server, and their names should not be changed. A global file was used for package management. The main packages used are tidyverse, which is a collection of R packages designed for data science analysis, and Shiny, which facilitates building interactive web apps directly from R. Finally, the last two R files contain support functions to start the analysis and scripts for calculating abnormalities, respectively.

**Fig. 14:**
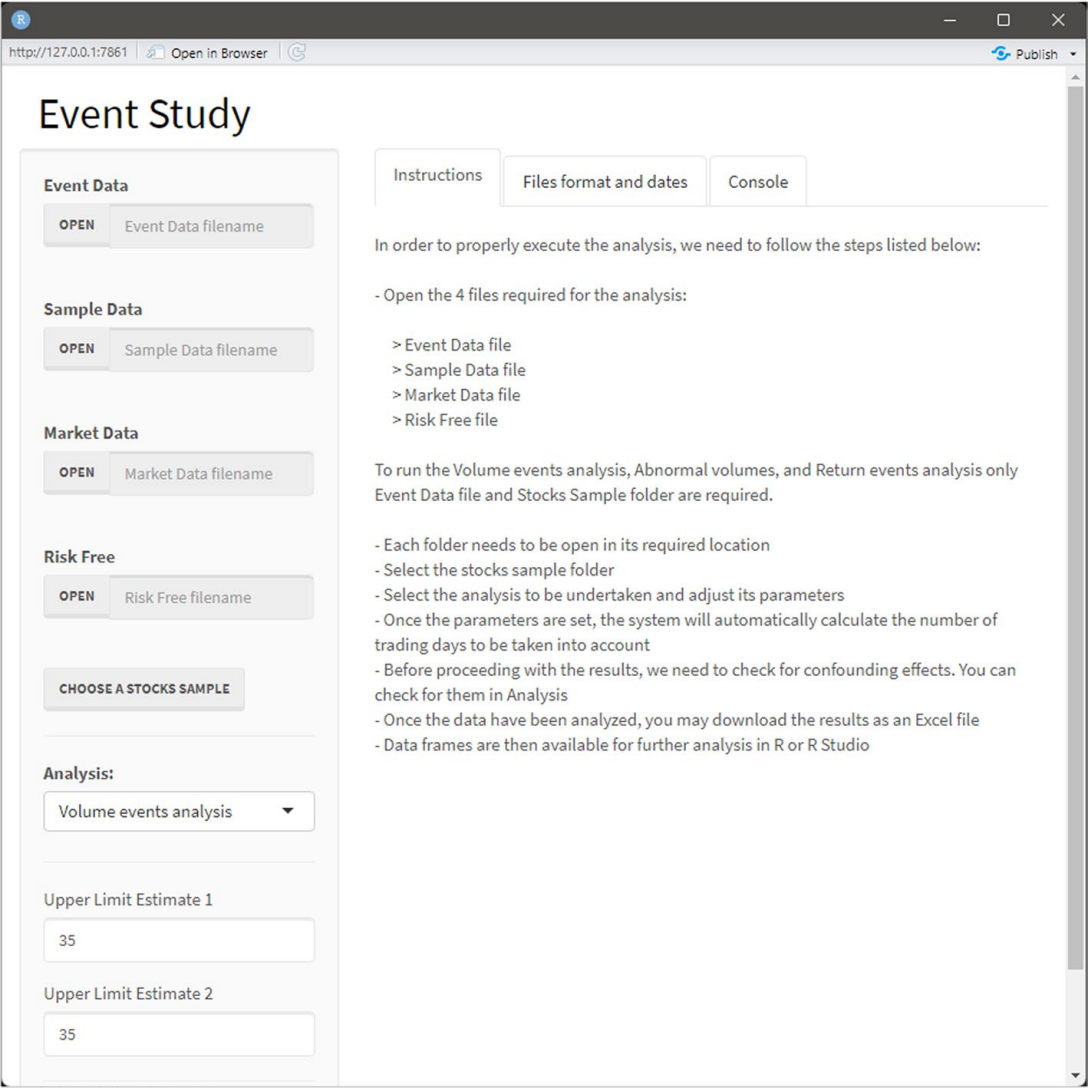
Dashboard interface. *Source*: Created by authors

**Fig. 15:**
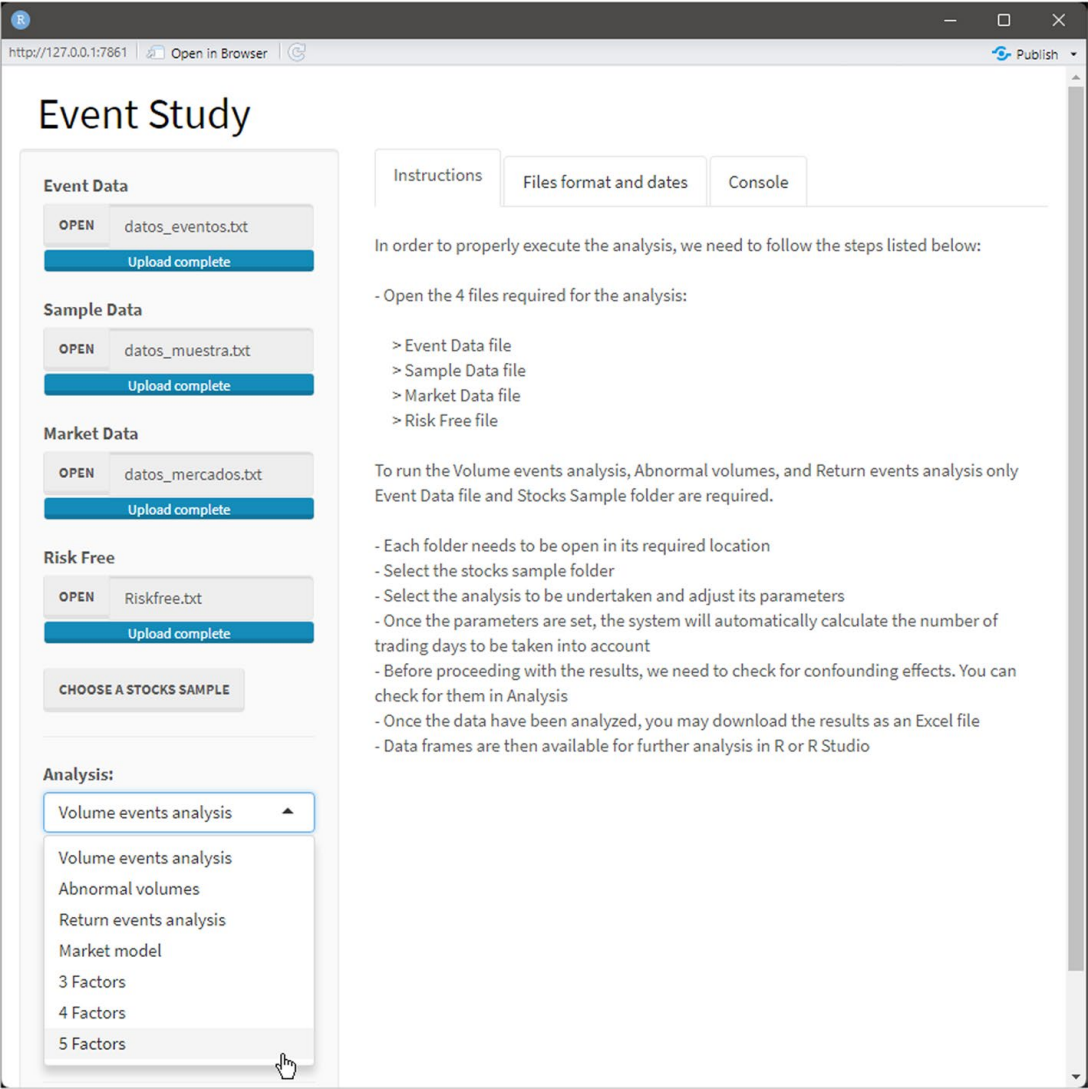
Drop-down menu. *Source*: Created by authors

When the script is executed, the dashboard opens, as shown in Fig. 14. The interface of the dashboard is divided into two parts: a lateral panel on the left side, where users can introduce inputs (e.g., the parameters of the event study, such as the upper limit of the estimation window), and a main panel on the right side, where the outputs are displayed. Among the inputs, the following are shown: uploading files (event, market, sample, risk-free data text files, and the stock sample folder), a drop-down menu to choose the type of analysis to be conducted and its parameters, and an analysis button to execute it (Fig. 15). The tool provides functions to perform volume event analysis, abnormal volume analysis, and return event analysis, and to estimate expected and abnormal returns using the market model—the French three- and five-factor models, and the Carhart four-factor model.

All text files uploaded to the interface need to be structured in a specific way, as shown in Table 3 for the DJIA index. Further details on text file specifications and common mistakes to be avoided can be found in “Appendix C”. A summary of this is also included in the dashboard; see the Files format and dates in Fig. 15.

**Table 3:** Text file structure

Text file	Number of columns	Columns names
Events data	3	COMPANY, DATE, MARKET
Market data	2	Date, DJ30
Sample data	14	COMPANY, TICKER, MARKET, YEAR(t), STARTING_PRICE_YEAR(t − 1), CLOSING_PRICE_YEAR(t − 1), MARKET_CAP(t − 1), TOTAL_EQUITY(t − 1), REVENUES(t − 1), COGS(t − 1), SG&A(t − 1), INTEREST_EXPENSE(t − 1), ASSETS(t − 1), ASSETS(t − 2)
Risk-free data	2	Date, RF
Companies’ files	3	Date, PX_LAST, PX_VOLUME

Source: Created by authors

The MARKET column content in the event data file should correspond to the second column name in the market data file depending on the index analyzed.

**Fig. 16:**
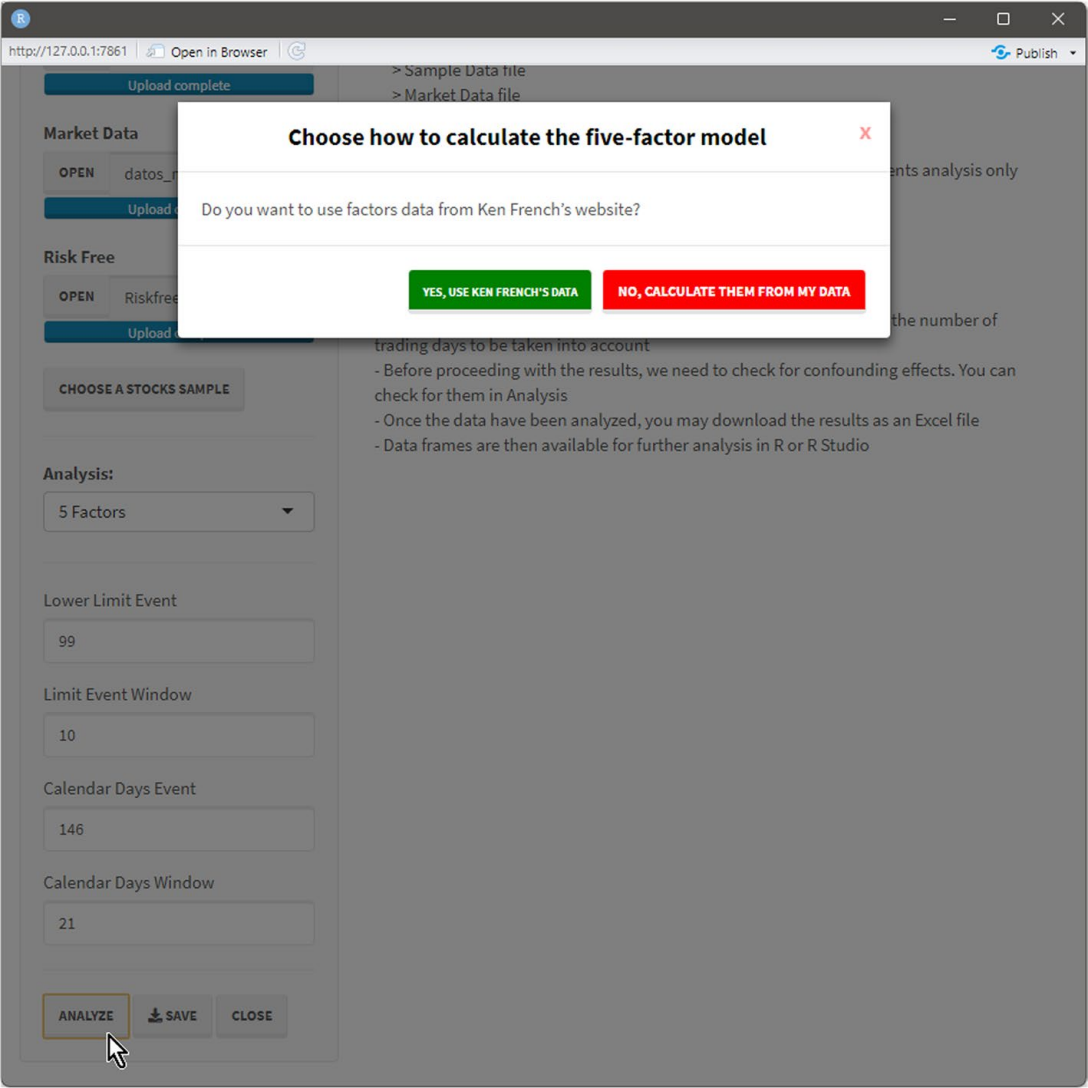
Market portfolio data source pop-up. *Source*: Created by authors

**Fig. 17:**
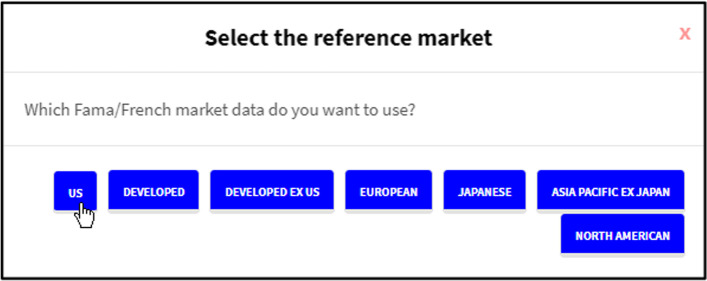
Kenneth R. French market portfolio selection pop-up. *Source*: Created by authors

Once the data files are loaded and the analysis to be performed is chosen, the user presses the Analyze command button to proceed with the calculations. When three-, four-, or five-factor models are chosen, the interface allows the selection of whether to use Kenneth R. French’s data or calculate the factors using the uploaded data (Fig. 16). If the first option is followed, a second pop-up appears (Fig. 17) to select a market portfolio.

**Fig. 18:**
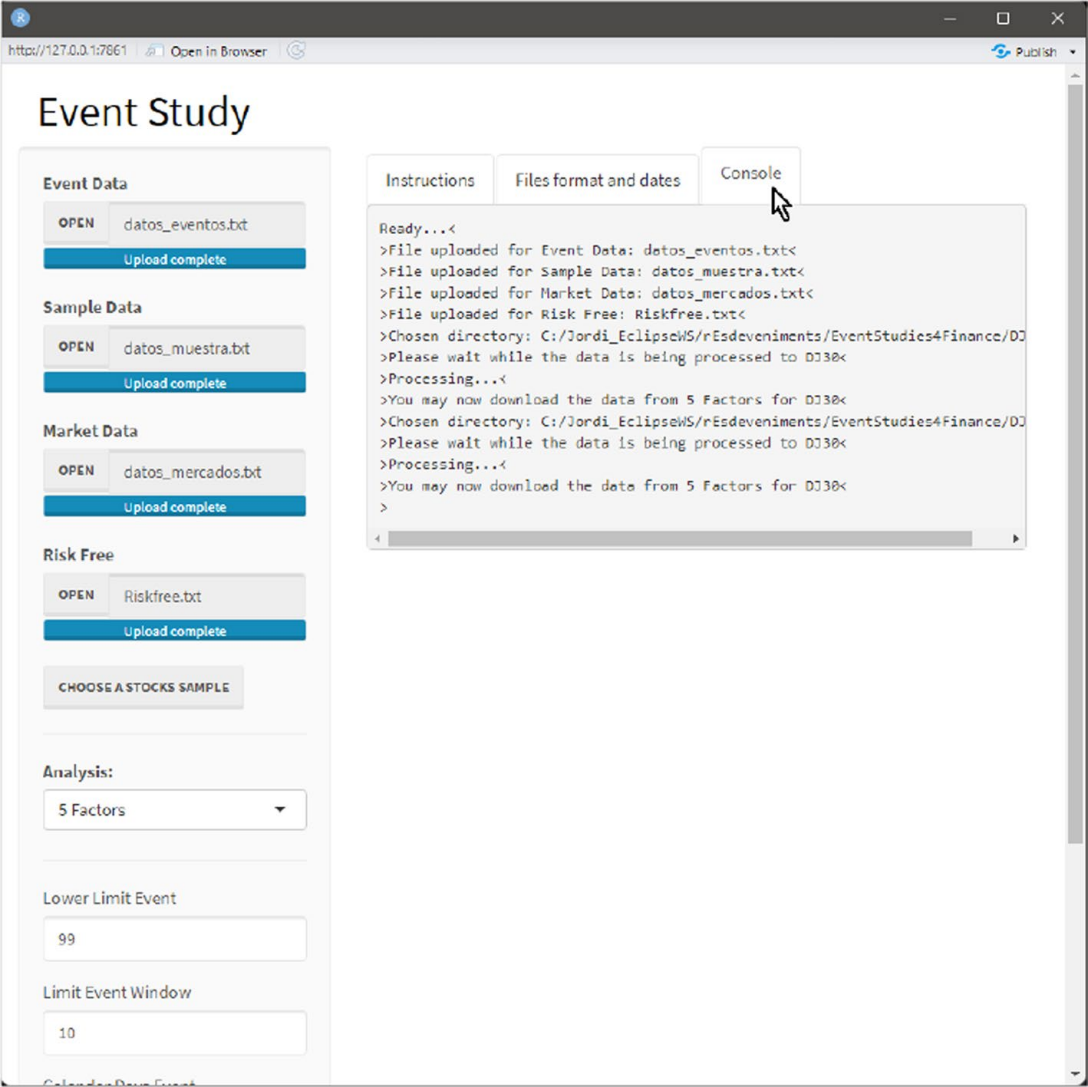
Console tab. *Source*: Created by authors

An instruction panel was displayed to facilitate an understanding of how the dashboard should be used. The third tab with the console, where process messages are produced, is shown in Fig. 18. Finally, there are two additional buttons at the bottom of the left panel. One allows the user to save the analysis developed internally as an MS Excel file, and the other allows the user to close the dashboard.

## Results and discussion

As the main outcome of this study, seven MS Excel files and six R files were generated to develop the analyses required for the event study methodologies using both tools. All these tools have been applied to the study of a single event, the announcement of the COVID-19 vaccine, to explore its effects on the DJ30 companies' stock prices. However, both the R tool and Excel calculations can be run for multiple events and companies.

**Fig. 19:**
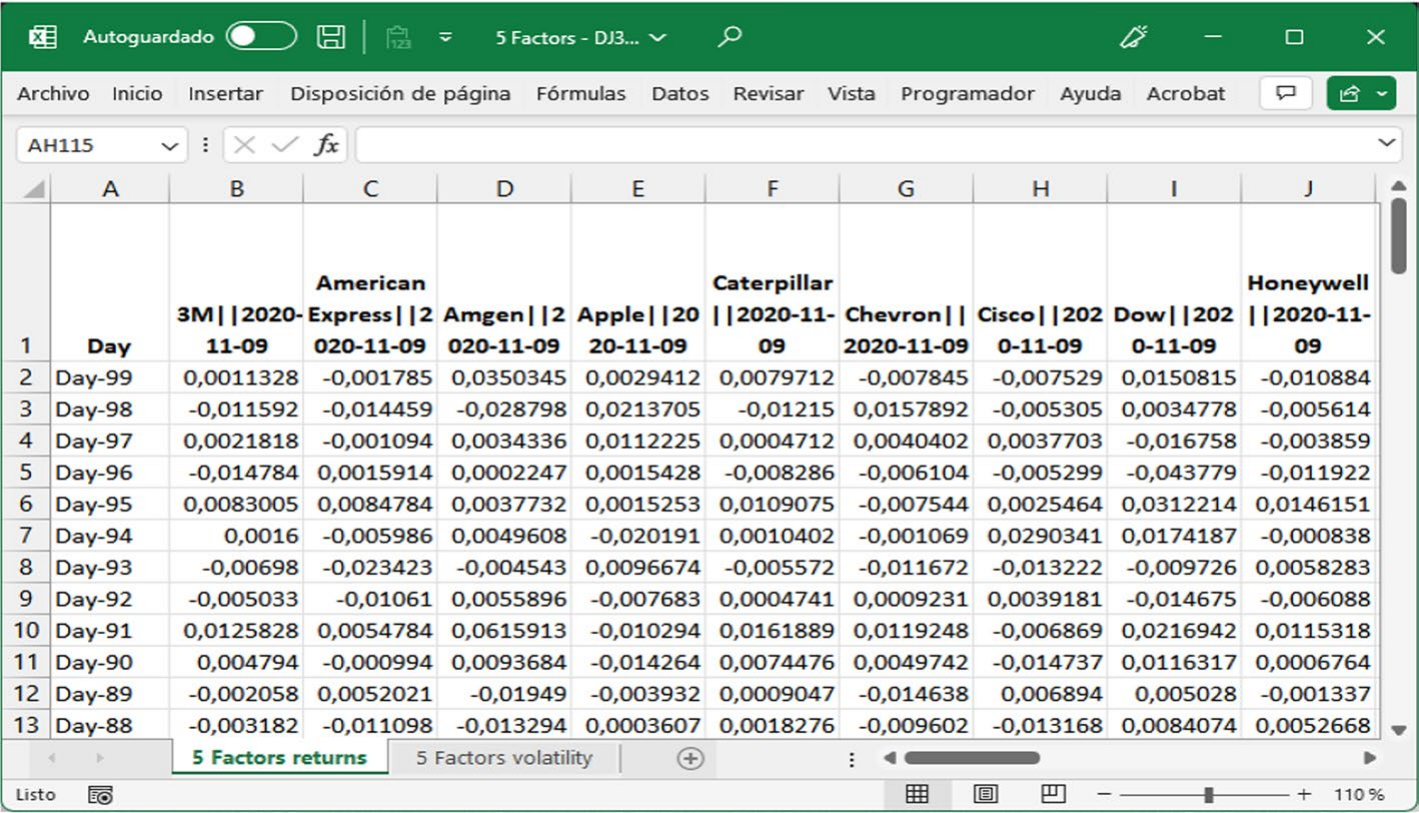
Five-factor Excel file downloaded, first columns. *Source*: Created by authors

**Fig. 20:**
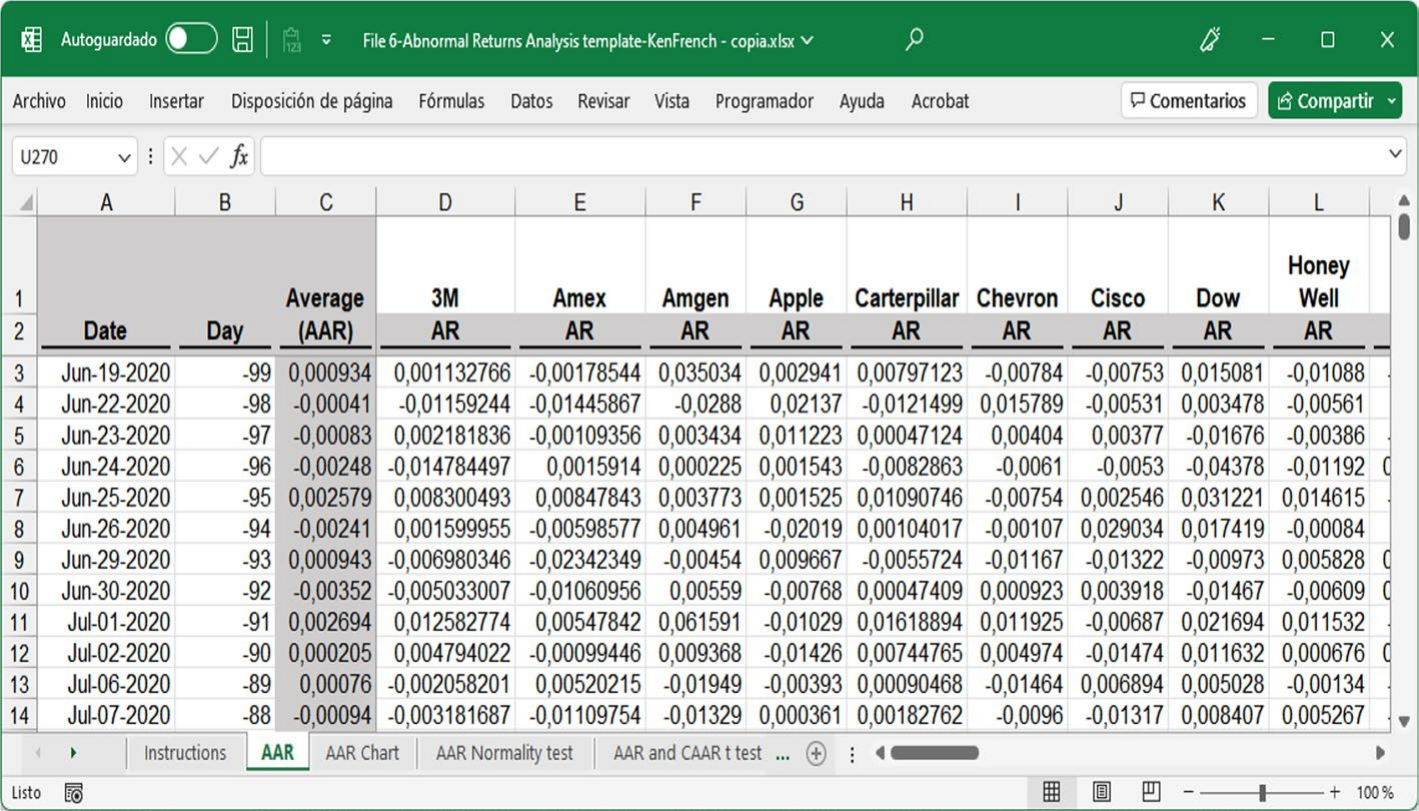
File 6-Abnormal Returns Analysis template, first columns. *Source*: Created by authors

The numerical results obtained in the analyses were the same, regardless of the tool used. A screenshot of the five-factor MS Excel file downloaded from the dashboard is shown in Fig. 19. For this analysis, two tables, including all the calculations performed, were obtained. The first corresponds to returns and the second corresponds to volatility. In both cases, the Fama–French five-factor model was used for the calculations by day and event. The AR results per company displayed in Fig. 19 are identical to those available in File 6-Abnormal Returns Analysis template.xlsx (Fig. 20), although the decimal positions and column orders are not equal. When calculating volatility and volume using the five-factor model or any other model, the same results were obtained.

Both files are equivalent as they contain the same numerical results, but not equal, as the MS Excel template (Fig. 20) contains all formulas and functions that can be consulted at any time, whereas the Excel file downloaded from the R tool interface contains numerical values only. These matching results validate the tools described above.

Once the equivalence between the numerical values obtained using each tool has been verified, an a posteriori graphical study summarizing the results of the event analysis can be performed, as shown at the end of the previous section.

Another important aspect of this study is the comparison of tools for the development and teaching of the event-analysis methodology. In this regard, the use of MS Excel or any other spreadsheet programme requires much more manual processing and is exposed to potential errors that are difficult to identify and correct. The use of R, particularly Shiny, enabled better reproducibility in this study. Despite these advantages, a major weakness, as previously noted, should be mentioned. When instructors or students use the Shiny dashboard instead of Excel templates, the MS Excel file downloaded from the R tool interface contains only numerical values, and no formulas or functions are shown. This means that while the templates can be used in the teaching process of the event study methodology—a learning tool in itself—the dashboard is mainly a shortcut to obtaining the results needed for further exploration but not a tool for teaching the initial calculations. Only if users have prior programming knowledge can the R tool be used to understand the calculations of the model, because the formulas can be found in the facilitated R scripts.

Another limitation of R is the required input file format. As described in Table 3, every text file must have a specified number of columns and specific column names for the dashboard to operate properly. Although this is common when using data analytics tools, the required formats do not match the files downloaded from commonly used financial databases, such as S&P Capital IQ. Consequently, input databases must be modified manually to fit the required formats.

A limitation of the MS Excel files we supply is precisely what led us to develop the R application. The sample size that can be processed manually with these files is relatively limited, not necessarily because of the capacity of the template in our context but because of the time it would take to adapt and perform the calculations for large samples. Therefore, we selected a relatively small sample of companies to show the sequence of calculations from the beginning to the end.

An additional limitation is related to the complexity of generating a single template that performs all computational event study steps automatically. Therefore, the entire process was divided into seven Excel files. Some of these can be used as semi-automatic templates and some as spreadsheets. In these templates or spreadsheets, repeated calculations must be executed manually (e.g., regression to obtain the betas) or results must be transferred manually from one file to another.

The next steps in our research are related to empirically evaluating both tools and to perform a usability test. For the first purpose, a teaching activity should be designed and conducted to gather students' results. A statistical analysis of the empirical results could be helpful in assessing the practical use of the tools presented. Usability analysis will provide a future measure of how usable these tools are and will facilitate an improvement in their effectiveness.

## Conclusions

The main outcomes of this research will benefit financial students, researchers, and professionals. On the one hand, in the EventStudies4Finance repository (Serrano and Cuadros 2022), seven MS Excel files developed to help instructors teach how to perform event studies are made available. They provide participants with an open set of templates. The set of MS Excel files allows the classification of portfolios and obtaining results using the Fama–French five-factor model. However, as it is not easy to find a free open-source tool to apply and teach event study methodology, an application using R code and the Shiny R package was developed to automatically perform an event study. The most popular estimation models can be used for this purpose by uploading the requested data files, as described in this study. These resources, along with the exercises provided, could be utilized to teach finance students how to conduct event studies and emphasize the significance of proficiency in the use of large data processing and analysis programs in financial education. Professionals may apply the same techniques to any market or portfolio of their choice to implement event-driven investment strategies.

The main advantage of the first outcome of this study, based on MS Excel, is that it provides a semi-automatic tool from which the calculations to complete an event study analysis can be identified. To teach event study methodology, students need to understand how to obtain an expected return, and MS Excel allows the visualization of the equations used. Another advantage of using MS Excel is that most students are familiar with the tool, and it is easy to access. However, reusing templates is subject to potential errors and manual changes should be performed. It should be highlighted that, with MS Excel, the sequence followed for the calculations was not explicit.

Some of these drawbacks were overcome when using the second tool, which is based on the R programming language. Furthermore, as the dashboard was created, the instructors and students did not require prior programming skills. No changes need to be made even if there is a large amount of data to analyze and numerous companies to classify. Additionally, a wide range of models and calculations were implemented. Nevertheless, the use of the second tool does not allow users without programming knowledge to examine how internal calculations and their sequences are performed in the development of the study of events, which remains a black box.

## Data Availability

The data and files that support the findings of this study are available at: https://github.com/vanessaserrano/EventStudies4Finance

## Appendix

### Appendix A: Accounting data used and sample descriptive statistics

**Table:**

Company	Market Capitalisation (t − 1)	Equity (t − 1)	Revenue (t − 1)	SG&A Exp. (t-1)	Interest Expense (t − 1)	Cost of Goods Sold (t − 1)	Total Assets (t − 1)	Total Assets (t − 2)
3 M Company	101,450.40	10,126.00	32,136.00	6,390.00	− 448.00	16,736.00	44,659.00	36,500.00
American Express Company	101,866.50	23,071.00	39,983.00	13,051.00	NA	12,661.00	198,321.00	188,602.00
Amgen Inc	143,239.80	9,673.00	23,362.00	5,150.00	− 1,289.00	4,356.00	59,707.00	66,416.00
Apple Inc	1,304,764.80	89,531.00	267,683.00	18,659.00	− 3,471.00	166,105.00	340,618.00	373,719.00
Caterpillar Inc	81,616.60	14,629.00	53,800.00	4,796.00	− 421.00	36,630.00	78,453.00	78,509.00
Chevron Corporation	227,869.00	145,208.00	139,865.00	26,590.00	− 798.00	80,113.00	237,428.00	253,863.00
Cisco Systems, Inc	203,458.90	35,533.00	51,550.00	11,711.00	− 751.00	18,575.00	90,426.00	102,462.00
Dow Inc	40,582.10	14,094.00	42,951.00	1,385.00	− 933.00	36,621.00	60,524.00	83,699.00
Honeywell International Inc	126,472.40	18,713.00	36,709.00	4,866.00	− 357.00	24,339.00	58,679.00	57,773.00
Intel Corporation	260,347.50	77,504.00	71,965.00	6,350.00	− 489.00	29,825.00	136,524.00	127,963.00
International Business Machines Corporation	118,710.80	20,985.00	77,147.00	20,382.00	− 1,344.00	39,754.00	152,186.00	123,382.00
Johnson & Johnson	383,911.20	59,471.00	82,059.00	22,178.00	− 318.00	27,456.00	157,728.00	152,954.00
JPMorgan Chase & Co	437,160.10	261,330.00	110,041.00	60,410.00	NA	NA	2,687,379.00	2,622,532.00
McDonald's Corporation	148,818.80	− 8,210.30	21,076.50	2,229.40	− 1,121.90	9,961.20	47,510.80	32,811.20
Merck & Co., Inc	231,557.30	26,001.00	46,840.00	9,863.00	− 893.00	13,156.00	84,397.00	82,637.00
Microsoft Corporation	1,203,062.60	110,109.00	134,249.00	23,583.00	− 2,632.00	43,346.00	282,794.00	258,859.00
NIKE, Inc	158,150.20	9,351.00	40,781.00	13,149.00	− 128.00	22,394.00	26,602.00	22,677.00
Salesforce.com, inc	144,261.70	33,885.00	17,098.00	9,594.00	− 131.00	4,235.00	55,126.00	30,737.00
The Boeing Company	183,334.90	− 8,300.00	76,559.00	3,642.00	− 722.00	71,738.00	133,625.00	117,359.00
The Coca-Cola Company	237,146.60	21,098.00	37,266.00	12,011.00	− 946.00	14,619.00	86,381.00	83,216.00
The Goldman Sachs Group, Inc	81,415.20	91,978.00	35,481.00	15,437.00	NA	4,419.00	992,968.00	931,796.00
The Home Depot, Inc	238,215.70	− 3,116.00	110,225.00	19,740.00	− 1,201.00	72,653.00	51,236.00	44,003.00
The Procter & Gamble Company	311,477.10	45,908.00	69,594.00	19,482.00	− 450.00	34,436.00	111,723.00	123,687.00
The Travelers Companies, Inc	35,348.50	25,943.00	31,581.00	4,365.00	− 344.00	23,734.00	110,122.00	104,233.00
The Walt Disney Company	260,680.90	103,802.00	75,125.00	12,812.00	− 1,445.00	46,033.00	200,948.00	99,941.00
UnitedHealth Group Incorporated	278,521.00	62,162.00	242,155.00	35,193.00	− 1,704.00	184,557.00	173,889.00	152,221.00
Verizon Communications Inc	253,937.20	62,835.00	131,868.00	28,939.00	− 4,730.00	54,726.00	291,727.00	264,829.00
Visa Inc	404,885.20	35,270.00	23,525.00	6,387.00	− 499.00	729.00	74,781.00	71,655.00
Walgreens Boots Alliance, Inc	52,355.70	24,314.00	137,412.00	24,836.00	− 684.00	107,715.00	90,807.00	69,941.00
Walmart Inc	337,169.90	81,552.00	523,964.00	107,891.00	− 2,599.00	394,605.00	236,495.00	219,295.00
Minimum	35,348.50	− 8,300.00	17,098.00	1,385.00	− 4,730.00	729.00	26,602.00	22,677.00
Median	215,663.95	29,943.00	61,697.00	12,931.50	− 798.00	29,825.00	110,922.50	103,347.50
Average	269,726.29	49,814.99	92,801.68	18,369.05	− 1,142.55	55,042.32	245,125.46	232,609.04
Maximum	1,304,764.80	261,330.00	523,964.00	107,891.00	− 128.00	394,605.00	2,687,379.00	2,622,532.00
Stan. Desv	288,266.71	55,281.97	101,626.06	20,843.12	1,078.08	79,061.80	494,170.71	481,975.58

Source: S&P Capital IQ

Data in USD Millions. Information referred as t − 1 corresponds to 31/12/2019 and t − 2 to 31/12/2018

### Appendix B: Abnormal returns

Source: Created by authors.

### Appendix C: Text files specifications and common mistakes

As already indicated in Table 3, running an Event Study in the R tool will require the preparation of the text files described there: Events data, Market data, Sample data, Risk.free and Companies' files. Table 3 includes the number of columns and the column names. Further details are given below.

- All text files are UTF-8 encoded and use tabs for column separations.
- Strings are unquoted.
- Decimal character is a point.
- Windows-like end-of-line characters are expected after each line, including the last one.
- Header appears in the first line.
- Dates are formatted as dd/mm/yyyy, for instance 29/10/2020
- Year is formatted as yyyy, for
- instance 2020.
- Missing data should be coded as any of these options: "","#N/A","N/A","NULL","-" or "NA".
- When monetary values are included, no monetary symbol should be used.

Most common mistakes take place when failing to follow some of these specifications.

## Abbreviations

A: Aggressive
AR: Abnormal Return
AAR: Average Abnormal Return
AbsAAR: Absolute Value Average Abnormal Return
AAV: Average Abnormal Volume
CAAR: Cumulative Average Abnormal Return
B: Big
C: Conservative
CAbsAAR: Cumulative Absolute Value Abnormal Return
CAAV: Cumulative Average Abnormal Volume
CMA: Conservative minus Aggressive
DJIA: Dow Jones Industrial Average
H: High
L: Low
HML: High minus Low
R: Robust
S: Small
S/H: Small and High
S/L: Small and Low
S/M: Small and Medium
SMB: Small minus Big
RMW: Robust minus Weak
W: Weak

## Footnotes

1 https://www.eventstudytools.com/how-cite-eventstudytoolscom.

2 https://wrds-www.wharton.upenn.edu/pages/analytics/.

3 http://breese7160.tulane.edu/event-study/.

4 https://github.com/vanessaserrano/EventStudies4Finance/tree/FINI202211.
